# Elevated Maternal Folate Status and Changes in Maternal Prolactin, Placental Lactogen and Placental Growth Hormone Following Folic Acid Food Fortification: Evidence from Two Prospective Pregnancy Cohorts

**DOI:** 10.3390/nu15071553

**Published:** 2023-03-23

**Authors:** Tanja Jankovic-Karasoulos, Melanie D. Smith, Shalem Leemaqz, Jessica Williamson, Dylan McCullough, Anya L. Arthurs, Lauren A. Jones, Konstantinos Justin Bogias, Ben W. Mol, Julia Dalton, Gustaaf A. Dekker, Claire T. Roberts

**Affiliations:** 1 Flinders Health and Medical Research Institute, Flinders University, Adelaide, SA 5000, Australia; 2 Department of Obstetrics and Gynaecology, Monash University, Clayton, VIC 3800, Australia; 3 Lyell McEwin Hospital, Adelaide, SA 5112, Australia; 4 Lyell McEwin Hospital, The University of Adelaide, Adelaide, SA 5000, Australia

**Keywords:** folic acid, prolactin, human placental lactogen, placental growth hormone, pregnancy, obesity, gestational diabetes mellitus

## Abstract

Folic acid (FA) food fortification in Australia has resulted in a higher-than-expected intake of FA during pregnancy. High FA intake is associated with increased insulin resistance and gestational diabetes. We aimed to establish whether maternal one-carbon metabolism and hormones that regulate glucose homeostasis change in healthy pregnancies post-FA food fortification. Circulating folate, B12, homocysteine, prolactin (PRL), human placental lactogen (hPL) and placental growth hormone (GH2) were measured in early pregnancy maternal blood in women with uncomplicated pregnancies prior to (SCOPE: N = 604) and post (STOP: N = 711)-FA food fortification. FA food fortification resulted in 63% higher maternal folate. STOP women had lower hPL (33%) and GH2 (43%) after 10 weeks of gestation, but they had higher PRL (29%) and hPL (28%) after 16 weeks. FA supplementation during pregnancy increased maternal folate and reduced homocysteine but only in the SCOPE group, and it was associated with 54% higher PRL in SCOPE but 28% lower PRL in STOP. FA food fortification increased maternal folate status, but supplements no longer had an effect, thereby calling into question their utility. An altered secretion of hormones that regulate glucose homeostasis in pregnancy could place women post-fortification at an increased risk of insulin resistance and gestational diabetes, particularly for older women and those with obesity.

## 1. Introduction

Adequate maternal folate (vitamin B9) is essential for pregnancy health [1]. Folic acid (FA) is a synthetic form of folate that is used in pregnancy supplements and food fortification [2]. In Australia, folic acid (FA) supplementation, with a daily dose of 400 μg for at least one month prior to and during the first trimester of pregnancy, has been part of clinical guidelines for over 20 years and was proven to reduce the incidence of neural tube defects [3,4]. To ensure that all women of reproductive age have sufficient folate levels, including those who may conceive unintentionally and are unlikely to supplement, over 80 governments across the world implemented mandatory FA food fortification programs [5]. The Australian government implemented a FA fortification mandate for bread-making flour in 2009 [6]. The mandate was expected to increase daily FA intake by 100 μg. However, most pregnant women post-FA fortification mandate are exceeding the recommended total daily intake of 600 µg [7,8]. A recent systematic review of women taking FA supplements in countries with mandatory food fortification programs found that almost all women exceed the upper tolerable limit of FA (1000 μg/day) [7]. A possible explanation for the disparity between the expected and actual daily increase, at least in Australia, is that within a couple of years after the FA fortification mandate, the voluntary fortification of numerous food products with FA commenced, resulting in substantially higher intakes of FA than predicted from the fortification mandate alone. Given widespread FA food fortification and the fact that many pregnancy supplements available in Australia contain more than 400 µg FA, it is likely that pregnant women in Australia are indeed exceeding the recommended daily FA intake, with unknown repercussions for pregnancy health.

**Figure 1:**
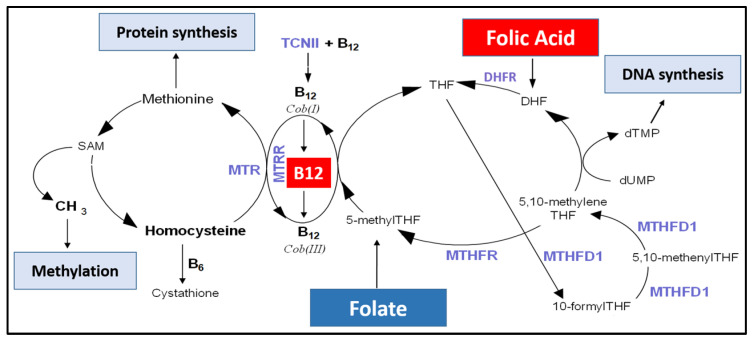
One-carbon metabolism: Overview of one-carbon metabolism. Folic acid (FA) is reduced via dihydrofolate reductase (DHFR) to dihydrofolate (DHF) and tetrahydrofolate (THF). Methylenetetrahydrofolate dehydrogenase (MTHFD1) converts THF to intermediate metabolites 10-formyltetrahydrofolate (10-formylTHF), 5,10-methenyltetrahydrofolate (5,10-methenylTHF) and 5,10-methylenetetrahydrofolate (5,10-methyleneTHF). 5,10-methyleneTHF can be used in the conversion of deoxyuridine monophosphate (dUMP) to deoxythymidine monophosphate (dTMP) via thymidylate synthase (TS) or to 5-methylTHF via methylenetetrahydrofolate reductase (MTHFR). 5-methylTHF is used for homocysteine remethylation to methionine, which is reliant on vitamin B12 (B12)-dependent methionine synthase (MTR). Methionine is converted to S-adenosylmethionine (SAM), a methyl donor in methylation reactions, and sequentially to S-adenosylhomocysteine (SAH), a substrate of homocysteine remethylation. Adapted from Williamson et al., 2022 [34].

FA is a metabolically inactive compound that undergoes sequential reductions to generate a biologically active metabolite 5-methyl tetrahydrofolate (5-methylTHF; Figure 1), the main form of folate found in plasma [9]. Various FA metabolites play key roles in one-carbon metabolism (1Cm), a complex set of reactions that form two vital biological cycles: folate and methionine (Figure 1). Folate metabolites donate carbon groups for nucleic acid biosynthesis and DNA repair (folate cycle) as well as methyl groups required for global methylation and protein synthesis (methionine cycle) [10,11,12]. The interconversion between different folate forms will favour either folate or methionine cycle, depending on cellular needs [13]. Given that circulating folate is readily taken up from maternal circulation by the placenta, [14,15] low or high FA intake and maternal folate status can affect placental methylation, gene expression and protein synthesis, development and function and, ultimately, pregnancy health [16,17,18,19,20,21,22]. We, and others, showed that maternal folate deficiency increases the risk of delivering small and low-birthweight babies and of preterm birth, and that FA supplementation reduces these risks [23,24,25,26,27]. However, the influence of maternal folate excess on pregnancy outcomes is largely unknown. Recent studies show that high FA intake can lead to saturation of certain enzymes in the 1C metabolic pathway, leading to an accumulation of unmetabolized FA (UMFA) [28,29,30]. Concerns were raised regarding adverse health effects due to UMFA, which can be detected in circulation with daily FA doses as low as 300–400 μg [31]. UMFA levels are higher in people who supplement with FA alone compared to combined FA and vitamin B12, an essential co-factor in 1Cm [32]. Despite its important role in FA metabolism, as well as its ability to reduce levels of pro-inflammatory homocysteine (high levels of which are associated with several pregnancy complications [33]) (Figure 1), B12 was not included in the Australian FA food fortification mandate. However, many food manufacturers voluntarily add B12 to various food products.

High FA/folate as well as a combination of high Folate:B12 and Hcy are associated with increased insulin resistance and risk of gestational diabetes mellitus (GDM) [35,36,37,38,39]. Interestingly, the incidence of GDM in Australia has tripled over a decade, from 5.4% in 2008 to 16.1% (43,100 births) in 2018, [40] coinciding with the Australian FA food fortification mandate and voluntary food fortification. It is important to note that the trajectory of the GDM rise started before and continued after the implementation of new 2015 GDM classification guidelines; thus, only a fraction of the increased GDM incidence can be attributed to changes in diagnostic criteria [41].

Findings from animal models suggest that high maternal FA intake leads to dysregulation of maternal and/or foetal one-carbon metabolism (1Cm) and lipid metabolism as well as increased insulin resistance and glucose intolerance, but the proposed mechanisms are lacking [42,43,44,45,46]. Although rising, the incidence of Type 2 diabetes in Australia is not on the same trajectory as GDM; hence, given their shared aetiology, it is likely that FA acts at least partially via the placenta, a transient organ unique to pregnancy that is an essential regulator of maternal insulin resistance and glucose transport to the foetus [47]. To control glucose homeostasis during pregnancy while maximizing glucose transport to the foetus, several placental peptide hormones are secreted into maternal circulation in addition to the pituitary- and decidua-released hormone prolactin [48,49]. Placental growth hormone variant (GH2) is secreted into maternal circulation to promote a state of mild insulin resistance. This is achieved by reducing insulin receptor expression and signalling, reducing insulin-sensitive glucose transporter (GLUT-4) in skeletal muscle and liver, and stimulating lipolysis to provide fatty acids for maternal energy use [49,50,51]. Increased insulin resistance is an essential maternal adaptation to pregnancy that ensures glucose availability for the foetus, as glucose is the preferred fuel for foetal growth and development [52]. Increased maternal insulin resistance calls for pancreatic beta cells to adapt and compensate by secreting more insulin [53]. Prolactin (PRL), secreted predominantly by the pituitary, and placental derived human placental lactogen (hPL) promote maternal pancreatic beta cell expansion and/or insulin secretion to control increased maternal glucose levels due to increased insulin resistance, thereby protecting against hyperglycaemia and the development of GDM [53]. PRL and hPL function via the PRL receptor located in pancreatic beta cells [54]. Interestingly, GH2 was also reported to increase pancreatic insulin secretion in human beta cells [55], although this role is less prominent than its role in promoting insulin resistance. Similarly, both PRL and hPL were reported to increase maternal insulin resistance [56], although their role in beta cell adaptation is greater.

Given that proper placental function relies on a balanced maternal nutrient supply [57,58,59,60], it is possible that changes in maternal micronutrient status (such as high folate or Folate:B12 imbalance) may impact aspects of placental function, such as hormone secretion, and ultimately affect pregnancy health. The aberrant secretion of hormones including PRL, hPL and GH2 discussed previously can place pregnant women at an increased risk of hyperglycaemia, GDM and potentially other adverse outcomes of pregnancy. To our knowledge, the association between FA and these hormones has not been explored.

Considering the steep GDM rise post-FA fortification in Australia, we aimed to better understand what changed in healthy pregnancies post-FA fortification that may contribute to an increased risk of GDM, particularly in those with known GDM risk factors such as increased maternal age and obesity. We aimed to determine if PRL and placental hormones hPL and GH2 are different in healthy pregnancies post- compared to pre-FA fortification. The work presented here explores powerful clinical and lifestyle databases and biobanks from women with uncomplicated pregnancies who were recruited to two large prospective pregnancy cohorts at the same hospital in Adelaide prior to (SCOPE) and post (STOP)-FA food fortification.

## 2. Materials and Methods

### 2.1. Study Population

**Figure 2:**
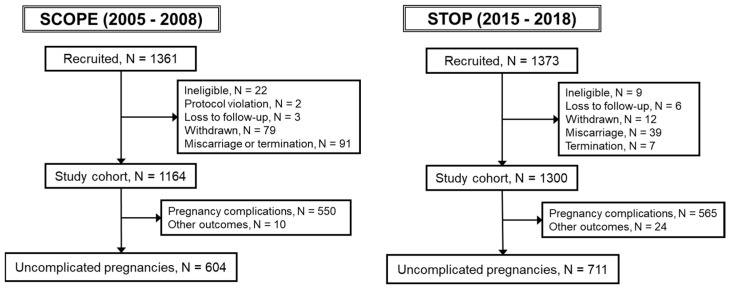
Study flow chart: Study exclusion criteria are shown for both SCOPE and STOP, with final totals of N = 604 and N = 711 participants, respectively, who had uncomplicated pregnancies.

This study used data from two prospective pregnancy cohorts that recruited women predominantly from the Lyell McEwin Hospital, Adelaide, South Australia. The Adelaide **Sc**reening f**o**r **P**regnancy **E**ndpoints (SCOPE; 2005–2008; Total N = 1164, Uncomplicated N = 604) cohort was part of a multi-centre international study of 5628 participants that aimed to develop screening tests to predict risk for pregnancy complications early in pregnancy before women became symptomatic. The **S**creening **T**ests to identify poor **O**utcomes of **P**regnancy (STOP; 2015–2018; Total N = 1300; Uncomplicated N = 711) cohort was established to validate screening tests for several pregnancy complications that were generated in SCOPE. The SCOPE cohort was recruited and gave birth prior to the 2009 FA fortification mandate, whereas the STOP cohort recruited women 6–9 years post-mandate. Nulliparous women with singleton pregnancies were recruited at 14–16 weeks of gestation in SCOPE and between 6–16 weeks in STOP. Women at high risk of pregnancy complications due to underlying medical history (e.g., type 1 or type 2 diabetes, hypertension, and related disorders), 3 or more previous miscarriages or terminations of pregnancy, those whose pregnancy was complicated by a known foetal anomaly and those who received interventions that may modify pregnancy outcomes (e.g., aspirin) were excluded from both studies. Women were also excluded if they were taking calcium (>1 g/d), eicosapentaenoic acid (≥2.7 g/d), vitamin C (>1000 mg/d) or vitamin E (>400 IU/d). The exclusion criteria for this study are outlined in Figure 2.

### 2.2. Data Collection

In this study, we compared the following baseline characteristics: maternal age, ethnicity, body mass index (BMI; calculated using maternal height and weight), demographic information with socio-economic index (SEI) as classified by using New Zealand’s criteria (scale of 10–90, with 10 representing the lowest SEI [61]), smoking status and micronutrient supplement use. Total daily doses of FA supplements were calculated from the quantity and formulation of tablets consumed, which included both discrete FA tablets and FA contained in multivitamin preparations. A non-fasting blood sample was taken at the time of recruitment and stored for downstream biochemical analyses that included circulating folate, vitamin B12, homocysteine (Hcy), red cell folate (RCF), PRL, hPL and GH2. 

An uncomplicated pregnancy was defined as a pregnancy in which no antenatal medical or obstetric complication had been diagnosed and as one which resulted in delivery of a healthy baby (birthweight customized centile >10th) at ≥37 weeks of gestation. Pregnancy and birth outcomes were ascertained from patient medical records by research midwives. Only women with a confirmed uncomplicated pregnancy were included in our data analyses.

### 2.3. Biochemical Measurements

Peripheral blood samples collected at 14–16 (SCOPE) and 6–16 (STOP) weeks of gestation were placed on ice, processed and stored at −80 °C for future analyses. Serum folate (nmol/L), B12 (pmol/L) and homocysteine (Hcy; μmol/L) quantifications were performed by Clinpath Laboratories, Adelaide, South Australia using chemiluminescent microparticle immunoassays on an Abbott Alinity automated immunoassay analyser. All samples were randomized to 96-well assay plates and measured at the same time to avoid cohort bias.

Red cell folate (RCF) was measured by the state pathology service (SA Pathology) using an ARCHITECT folate assay, Abbott Laboratories, Abbott Park, IL, USA per our previous publication [23]. RCF measurements were only available for the post-FA fortification cohort STOP. However, we previously published RCF data from 410 women at 12 weeks of gestation who were recruited at the same hospital at the same time as the SCOPE women (and thus, prior to FA food fortification), and we compared these data to those of the STOP women [23].

PRL (pg/mL; ALPCO, USA; Cat No: 25-PROHU-E01), hPL (μg/mL; Human Placental Lactogen ALPCO, USA; Cat No: 20-HPLHU-E01) and GH2 (pg/mL; Placenta-specific growth hormone variant; FineTest, China; Cat No: EH3133-96T) were measured in 465 SCOPE and 684 STOP women for whom we had available blood samples. Hormones were measured using Enzyme-Linked Immunosorbent Assays (ELISA) following the manufacturer’s instructions. All samples for the three hormone assays were diluted prior to use, and hormones were measured in duplicate by strictly following the recommended protocol guidelines. Optical density was measured using the SpectraMax iD5 plate reader. All measurements were performed using patient study numbers in a blinded manner. SCOPE and STOP samples were run on the same ELISA plates at the same time to avoid cohort bias.

### 2.4. Statistics

Descriptive statistics are reported for demographics and blood biochemistry measures by cohort. Means (SD), or medians (IQR) where appropriate, were used for continuous variables, and frequencies (%) were used for categorical variables. Linear regression was used to examine the association between blood biochemistry measures of one-carbon metabolism and of FA supplementation in SCOPE and STOP, adjusted for maternal age and BMI. An interaction term between blood biochemistry measures and the study was included to allow for non-constant effects across studies. Pre-specified post hoc contrasts comparing FA supplementation groups between and within studies were performed and adjusted for multiple comparisons. Data was log-transformed to approximate normality, and the ratios of geometric means (95% CI) are reported. Measures of red cell folate were compared between STOP and a separate pre-FA fortification cohort (N = 410) using the Mann–Whitney U test.

Serum hormone (PRL, hPL and GH2) concentrations were analysed using a linear mixed effects model, adjusting for gestational age at sampling. A random intercept for each participant was included to account for repeated measures from some participants in SCOPE who also had an earlier blood sample (at 12 ± 1 weeks of gestation) available. Separate models for maternal age and BMI were fitted, and an interaction term with the study was included. Pre-specified post hoc contrasts comparing each (maternal age and BMI) group between and within studies were performed and adjusted for multiple comparisons. Data were log-transformed to approximate normality, and the ratios of geometric means (95% CI) are reported.

Hormone concentrations (hPL and GH2) in media from human placental explants treated with different FA doses were analysed using linear regression with the post hoc Dunnett’s test and compared to 0nM FA treatment. Assumption checking was performed for all regression and linear mixed effects models. A two-sided alpha level of 0.05 was considered statistically significant. All analyses were performed using R version 4.2.1 (R Foundation for Statistical Computing, Vienna, Austria).

### 2.5. Ethics Approval and Patient Consent

SCOPE study ethics approval was obtained from the Central Northern Adelaide Health Service Human Research Ethics Committee (REC 1712/5/2008). STOP study ethics approval was obtained from the Women’s and Children’s Health Network Human Research Ethics Committee (HREC/14/WCHN/90). Ethics approval was obtained from Central Adelaide Local Health Network Human Research Ethics Committee to collect placentas from elective terminations of pregnancy at the Pregnancy Advisory Centre of The Queen Elizabeth Hospital in Adelaide (HREC/16/TQEH/33).

Pregnant nulliparous women attending their first antenatal clinic at the Lyell McEwin Hospital and Women’s and Children’s Hospital, Adelaide, South Australia, as well as women undergoing elective termination of pregnancy at The Queen Elizabeth Hospital, South Australia, were provided study participant information sheets. Written informed consent was obtained from all eligible women to participate in these studies.

SCOPE and STOP studies were registered with the Australian New Zealand Clinical Trial Registry (ACTRN12607000551493 and ACTRN12614000985684, respectively).

## 3. Results

### 3.1. Descriptive Characteristics

A total of 1164 women were recruited to SCOPE Adelaide. Of these, 604 (51.9%) women had an uncomplicated pregnancy (Figure 2). A total of 1300 women were recruited to STOP. Of these, 711 (54.7%) women had an uncomplicated pregnancy (Figure 2). Table 1 presents the maternal and pregnancy data for 604 SCOPE and 711 STOP women with an uncomplicated pregnancy. Compared to SCOPE, STOP women were older (Median (IQ): 23 y [20,21,22,23,24,25,26] vs. 25 y [22,23,24,25,26,27,28,29], *p* < 0.0001), had a higher SEI (Median (IQ): 24 [20,21,22,23,24,25,26,27,28,29,30] vs. 29 [22,23,24,25,26,27,28,29,30,31,32,33,34,35,36,37,38,39,40,41,42,43,44,45], *p* < 0.0001) and were less likely to be white (91.9% vs. 83.7%). There was no difference in early pregnancy BMI between the cohorts. Compared to SCOPE, fewer women smoked in early pregnancy in STOP (21.2% vs. 14.5%).

FA supplementation increased from 79.7% in SCOPE to 87.8% in STOP. Despite the 12.2% missing supplementation data in STOP (Table 1), indicating a possible underestimation, at least 62% of STOP women with an uncomplicated pregnancy used 800 μg or higher daily FA supplement tablets compared to 24.3% in SCOPE. Compared to SCOPE, vitamin B12 supplementation increased in STOP (53% vs. 71.6%; *p* < 0.0001; Table 1). 

Compared to SCOPE, STOP women had significantly higher serum folate (Median [IQ range]: 36.4 [28.5–43] vs. 40.5 [36.4–45.2] nmol/L; *p* < 0.0001), B12 (Median [IQ range]: 248 [195.5–316] vs. 277.5 [219–355] pmol/L; *p* < 0.0001) and homocysteine (Mean ± SD: 4.6 ± 1.1 μM in SCOPE vs. 5 ± 1.1 μM in STOP; *p* < 0.0001). Compared to SCOPE, STOP women with an uncomplicated pregnancy had a significantly higher Folate:B12 ratio (Median (IQR): 142.6 [104–207.4] vs. ±5.9 [115.7–222.4]; *p* = 0.0006; Table 1).

Regarding measured birth outcomes, gestational age at birth and birthweight were lower in STOP compared to SCOPE, but the mean customised birthweight centiles remained unchanged. The difference in gestational age is likely due to increased induction of labour before or at 40 weeks of gestation (SCOPE 22.2% vs. STOP 41.6%, *p* < 0.0001).

### 3.2. One-Carbon Metabolism Components in Early Pregnancy in Response to FA Supplementation

FA supplementation was divided into four categories: None; ≤400 μg; >400 to <800 μg; ≥800 μg. We used 400 μg and 800 μg as guides because in Australia, 400 μg is the recommended daily supplementation dose in pregnancy, and the most popular pregnancy supplement in Australia (Elevit, Bayer) has 800 μg per tablet. Table 2 presents maternal one-carbon metabolism blood biochemistry data (actual non-adjusted measurements) in early pregnancy for SCOPE and STOP women stratified by FA supplementation. SCOPE FA supplementation data was available for all 604 study participants, whereas STOP FA supplementation data was available for 624 of the 711 participants.

**Serum folate** was higher in SCOPE women who supplemented with FA compared to those who did not supplement. SCOPE women who supplemented with ≤400, >400 to <800 and ≥800 μg daily FA had 37% (ratio of geometric means (95% CI): 1.37 (1.09, 1.72) *p* = 0.0006), 53% (mean ratio (95% CI): 1.53 (1.30, 1.80) *p* < 0.0001) and 61% (mean ratio (95% CI): 1.61 (1.34, 1.93) *p* < 0.0001) higher median serum folate, respectively, than women who did not supplement with FA. In STOP women, however, FA supplementation during pregnancy did not result in higher serum folate concentrations. 

Although there was no difference in serum folate levels between the supplementation groups in STOP, STOP women who did not supplement with FA had 63% higher mean serum folate (Median (IQ): 38.9 [34.3–43.2]) compared to SCOPE women who did not supplement (Median (IQ): 27.1 [18.5–34.3]) (mean ratio (95% CI): 1.63 [1.28, 2.08]; *p* < 0.0001). There were no significant differences in serum folate levels between SCOPE and STOP for other FA supplementation categories.

**Serum Vitamin B12** was not statistically different between supplementation groups in SCOPE or STOP.

The **Folate:B12** ratio in SCOPE was 37% (mean ratio (95% CI): 1.37 [1.12, 1.66] *p* < 0.0001) and 43% (mean ratio (95% CI): 1.43 [1.15, 1.78] *p* < 0.0001) higher in women who supplemented with >400 to <800 μg and ≥800 μg FA, respectively, compared to women who did not supplement. No difference between Folate:B12 ratios was observed between FA supplementation categories in STOP. Compared to SCOPE, the overall Folate:B12 ratio was higher in STOP (Median (IQR): 142.6 [104–207.4] vs. 156 [115.7–222.4], *p* = 0.0006). Compared to SCOPE women who did not supplement with FA, STOP women who did not supplement had 37% higher Folate:B12 (ratio of GM (95% CI): 1.37 [1.02, 1.84] *p* = 0.02).

**Serum Hcy** concentrations were lower in SCOPE women who supplemented with FA daily compared to women who did not supplement (12% with ≤400 μg, *p* = 0.008; 11% with >400 to <800 μg, *p* = 0.0003; and 15% with ≥800 μg, *p* < 0.0001) (Table 2). Hcy was not different between FA supplementation groups in STOP. Interestingly, STOP women who supplemented with higher doses of FA, specifically those in the >400 to <800 μg and ≥800 μg groups, had 17% (ratio of GM (95% CI): 1.17 [1.09, 1.26] *p* < 0.0004) and 12% (ratio of GM (95% CI): 1.12 [1.05, 1.20] *p* < 0.0001) higher Hcy, respectively, compared to the same supplementation categories in SCOPE (Table 2).

**Figure 3:**
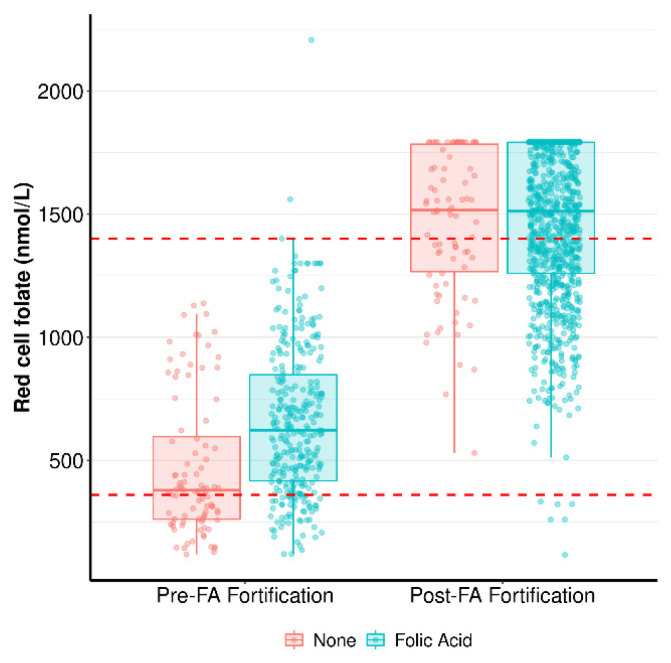
Red cell folate concentration in pregnant women prior to and post-FA food fortification: Data in the pre- and post-FA fortification categories are presented as medians (IQ range) and divided into two groups: women who did not take FA supplements (None, in red) and women who took FA supplements in the first trimester of pregnancy (Folic Acid, in green). The RCPA RCF reference range (360–1400 nmol/L) is represented by red dotted lines. The assay’s upper limit of detection is 1790 nmol/L. Measures of red cell folate were compared between STOP (N = 711) and a separate pre-FA fortification cohort (not SCOPE, N = 410) using the Mann–Whitney U test.

**Red cell folate (RCF)** data was available for STOP women but not for SCOPE. Therefore, we could not directly compare differences in RCF concentrations between SCOPE and STOP. However, we did have RCF data for 410 pregnant women at 12 weeks of gestation who were recruited at the same time and at the same hospital as SCOPE women, enabling a comparison of RCF levels prior to (N = 410) and after (STOP, N = 711) the FA food fortification mandate (Figure 3). Prior to the implementation of FA food fortification, women who supplemented with FA during early pregnancy had significantly higher RCF compared to women who did not supplement (Median (IQR): 623 nmol/L [417–848] vs. 379 nmol/L [261.2–596.5]; *p* < 0.0001). Although many women in the pre-FA food fortification cohort were folate-deficient, the median RCF was within the Royal College of Pathologists of Australasia (RCPA)’s reference range for normal RCF (shown by dashed red lines in Figure 3), regardless of whether women supplemented with FA during pregnancy. Following FA food fortification (STOP), FA supplementation during pregnancy had no effect on RCF (Median (IQR): no FA supplement: 1516 nmol/L [1266–1784] vs. FA supplement: 1512 nmol/L [1259–1792]; *p* = 0.9). 

Compared to women pre-FA fortification, STOP women had significantly higher RCF, regardless of whether they supplemented with FA during pregnancy or not (No FA supplement: pre-FA fortification Median (IQR): 379 nmol/L [261.2–596.5] vs. STOP 1516 nmol/L [1266–1784], *p* < 0.0001; FA supplement: pre-FA fortification 623 nmol/L [417–848] vs. STOP 1512 nmol/L [1259–1792], *p* < 0.0001). All STOP women, regardless of FA supplementation, had median RCF levels above the RCPA reference range for normal RCF. STOP concentration medians are, in fact, a conservative estimate, as many women post-FA fortification had RCF levels at the upper limit of assay detection (1790 nmol/L). Of the 711 STOP women with an uncomplicated pregnancy, only 6 were RCF-deficient.

### 3.3. Pregnancy Hormones

#### 3.3.1. Gestational Modelling

**Figure 4:**
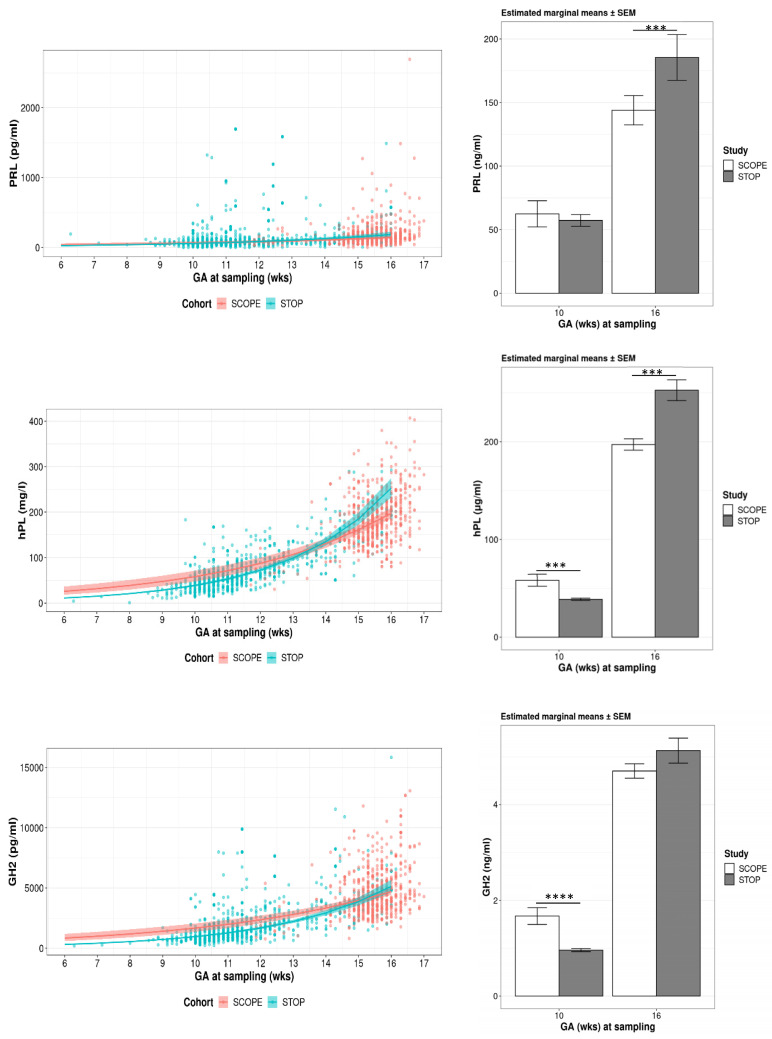
Serum hormone concentrations: PRL, hPL and GH2 concentrations across gestation are shown for SCOPE (N = 465) and STOP (N = 684) women with uncomplicated pregnancies. Estimated marginal means (±SEM) for SCOPE and STOP women at 10 weeks and 16 weeks of gestation are presented. Serum hormone (PRL, hPL and GH2) concentrations were analysed using a linear mixed effects model, adjusting for gestational age at sampling. Significance is indicated with asterisks (**** *p* < 0.0001 and *** *p* < 0.001).

PRL, hPL and GH2 were measured at 14–16 weeks of gestation in SCOPE and 6–16 weeks in STOP. Placental hormone secretion increases with gestation; hence, hormone data were adjusted for gestational age at the time of sampling using blood samples that were taken from the same SCOPE patients (N = 22) at both 11–13 and 14–16 weeks of gestation. Model fitting was performed to estimate changes in each hormone across early gestation (Supplementary Figure S1). Regression lines between 11–13 and 14–16 weeks of gestation samples show a clear separation (Supplementary Figure S1), supporting the need to account for gestational age at sampling in order to more accurately compare hormone levels between SCOPE and STOP women. As such, both 11–13 and 14–16 weeks of gestation samples from SCOPE were included as repeated measures in linear mixed effects models to generate a more accurate mean estimation across gestation for both cohorts and to account for the within and between-subject variances that are presented in Figure 4.

#### 3.3.2. Estimated Means for PRL, hPL and GH2

Estimated mean PRL, hPL and GH2 concentrations were plotted across early gestation between weeks 6 and 16 for SCOPE and STOP women (Figure 4), accounting for gestational age at blood sampling. Data presented are for N = 465 SCOPE and N = 684 STOP women with uncomplicated pregnancies for whom we had blood samples available. We calculated the estimated marginal means of each hormone at 10 weeks of gestation, at the time of onset of maternal blood flow to the placenta, and at 16 weeks, the latest time point of sampling in SCOPE and a time at which the second trimester’s exponential rise in hPL and GH2 is underway. Compared to SCOPE, at 10 weeks of gestation, estimated hPL and GH2 levels were significantly lower in STOP women (hPL −33%; 58.2 ± 6 vs. 38.8 ± 1.1 μg/mL, *p* < 0.0001 and GH2 −43%; 1.67 ± 0.17 vs. 0.96 ± 0.03 ng/mL, *p* < 0.0001), whereas at 16 weeks, STOP women had 29% higher circulating PRL and 28% higher hPL (185.5 ± 18 vs. 144 ± 11.5 ng/mL, *p* = 0.0004 and 253 ± 10.7 vs. 197 ± 5.8 μg/mL, *p* < 0.0001, respectively) (Figure 4). The level of GH2 at 16 weeks was not different between SCOPE and STOP (Ratio of geometric means (95% CI): 1.09 [0.99, 1.20]; *p* = 0.08).

#### 3.3.3. PRL, hPL and GH2 Concentrations Relative to FA Supplementation

**Figure 5:**
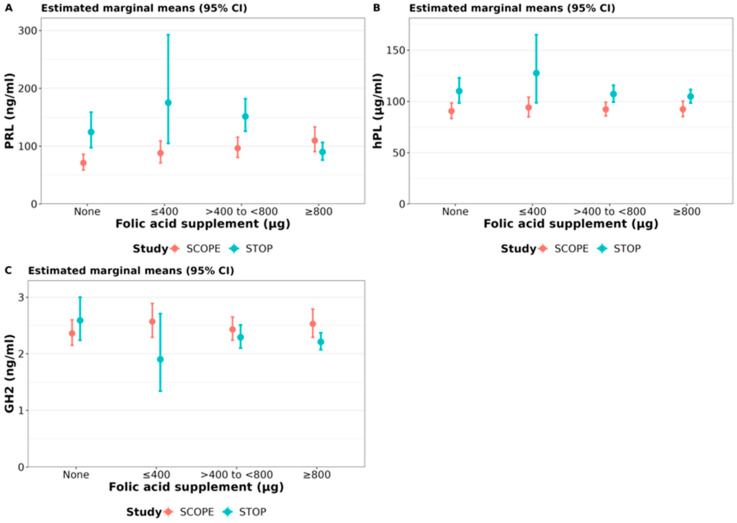
Serum hormone concentrations stratified by FA supplementation: Estimated marginal means for (**A**) prolactin (PRL), (**B**) human placental lactogen (hPL) and (**C**) placental growth hormone (GH2) in SCOPE (14–16 weeks of gestation) and STOP (6–16 weeks) women are stratified by four different FA supplementation categories (SCOPE N = 465; STOP N = 624).

To determine if there is an association between FA intake and pregnancy hormones, we analysed hormone concentrations (estimated marginal means) according to FA supplementation categories (Table 3 and Figure 5). Post hoc analyses compared hormone concentrations between different FA supplementation groups within each cohort, and the same FA supplementation groups between SCOPE and STOP. 

**Prolactin (PRL):** SCOPE women who supplemented daily with any dose of FA had significantly higher PRL concentrations than women who did not supplement (≤400 μg: +24% [95% CI: 0 to 54%] *p* = 0.05; >400 to <800 μg: +36% [95% CI: 14 to 62%] *p* < 0.0001; ≥800 μg: +54% [95% CI: 26 to 89%] *p* < 0.0001). The FA supplementation dose response observed in SCOPE was not present in STOP participants. STOP women who supplemented with the highest daily dose (≥800 μg) FA had: 28% (95% CI: 6 to 44%) lower PRL than women who did not supplement (*p* = 0.009); 49% (95% CI: 2 to 73%) lower PRL than women who supplemented with ≤400 μg (*p* = 0.04); 41% (95% CI: 31 to 49%) lower PRL than women who supplemented with >400 to <800 μg FA (*p* < 0.0001) (Table 3 and Figure 5).

Compared to SCOPE, STOP women had significantly higher PRL for each FA supplementation group (None: +75%, *p* < 0.0001; ≤400 μg: +99%, *p* = 0.01 and >400 to <800 μg: +57%, *p* < 0.0001), except for women who supplemented with the highest daily FA dose of ≥800 μg, who had 18% (*p* = 0.02) lower PRL than women in the same supplementation category in SCOPE (Table 3 and Figure 5).

**Human Placental lactogen (hPL):** hPL concentrations were not significantly different between daily FA supplementation categories in SCOPE nor in STOP. However, hPL concentrations were significantly higher in STOP compared to SCOPE women for all supplementation categories (None: 22% [95% CI: 7 to 38%] *p* = 0.003; ≤400 μg: 36% [95% CI: 3 to 78%] *p* = 0.03; >400 to <800 μg: 16% [95% CI: 6 to 27%] *p* = 0.002; ≥800 μg: 13% [95% CI: 4 to 24%] *p* = 0.007) (Table 3 and Figure 5).

**Growth hormone variant (GH2):** Like hPL, GH2 concentrations were not significantly different between daily FA supplementation categories in SCOPE nor in STOP. However, compared to SCOPE, in STOP women who supplemented with ≥800 μg, GH2 was 13% lower (95% CI: 2 to 22%, *p* = 0.02) (Table 3 and Figure 5).

#### 3.3.4. PRL, hPL and GH2 Concentrations Relative to Blood 1C Metabolism Biochemistry

To identify relationships between maternal 1C metabolism variables, including serum folate, B12 and Hcy and pregnancy hormones, we analysed hormone concentrations according to blood biochemistry data for both SCOPE and STOP. Given that there was no study effect when responses for each cohort were compared, SCOPE and STOP samples were combined for these analyses. We also assessed the association between RCF and pregnancy hormones in STOP.

Serum folate and RCF were not associated with any of the hormones studied. However, increased B12 was associated with increased PRL, hPL and GH2. Every 100 unit increase in B12 was associated with a 5% increase in PRL (95% CI: 2 to 9%), a 2% increase in hPL (95% CI: 0 to 4%) and a 3% increase in GH2 (95% CI: 1 to 5%) (Table 4). Interestingly, increased Folate:B12 ratios were associated with reduced GH2. Every 100 unit increase in Folate:B12 ratio was associated with a 2% reduction in GH2 (95% CI: 1 to 4%) (Table 4). Increased Hcy was associated with reduced PRL. Every one unit of increase in Hcy was associated with a 7% reduction in PRL (95% CI: 3 to 10%) (Table 4).

#### 3.3.5. PRL, hPL and GH2 Concentrations Relative to Maternal Age and/or BMI

Maternal age (≥35y) and obesity (BMI ≥ 30 kg/m^2^) are risk factors for several pregnancy complications, including increased insulin resistance and GDM. We analysed PRL, hPL and GH2 concentrations with respect to maternal age and obesity in SCOPE to determine if hormone concentrations are different in older women or those with obesity, or with a combination of the two in healthy pregnancy. We then assessed the same in STOP to determine whether maternal age and obesity alter these hormones in healthy pregnancies post-FA fortification. For these analyses, women with a healthy BMI (20–24.9 kg/m^2^) are referred to as ‘non-obese’, and their hormones are compared to those of women with obesity (BMI ≥ 30 kg/m^2^).

**Maternal age:** Maternal age did not influence PRL, hPL or GH2 in SCOPE. However, in STOP, women ≥35y had 31% lower PRL compared to women <35y (95% CI: 13 to 45%, *p* = 0.002; Table 5). Interestingly, STOP women ≥35y had higher serum folate compared to STOP women < 35y (Median [IQ range]: 44 [41.2–98] vs. 40.4 [36.3–44.9] nmol/L; *p* < 0.001), whereas in SCOPE, serum folate levels did not differ between the two age groups.

**Maternal obesity:** SCOPE women with obesity had lower PRL (−19%, [95% CI: 2 to 33%] *p* = 0.02), hPL (−21% [95% CI: 12 to 28%] *p* < 0.0001) and GH2 (−27%, [95% CI: 17 to 36%] *p* < 0.0001) compared to non-obese women (Table 6). In STOP, obese women had 30% lower PRL (95% CI: 18 to 41%, *p* < 0.0001) and 23% lower hPL (95% CI: 16 to 29%, *p* < 0.0001) compared to non-obese women. GH2 was not affected by obesity in STOP. Serum folate was not different between obese and non-obese groups in SCOPE nor in STOP.

**Interactive effects of maternal age and obesity:**

**Figure 6:**
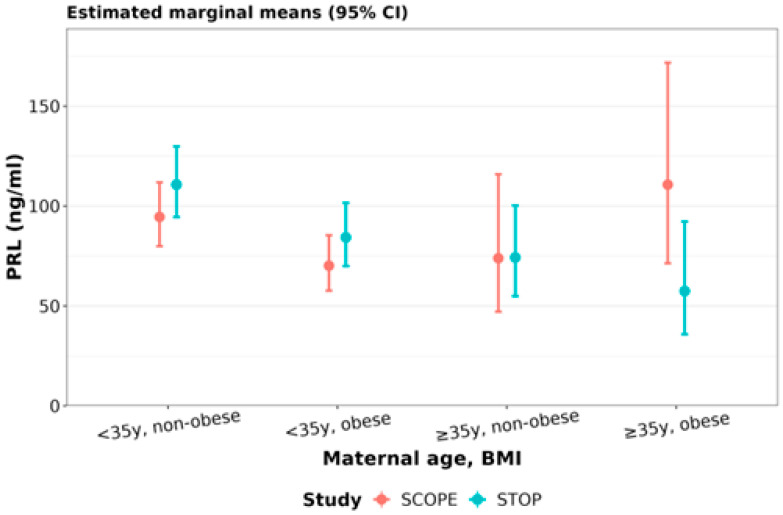
Prolactin concentrations stratified by maternal age and BMI: PRL in early gestation SCOPE (N = 465) and STOP (N = 684) women was stratified by maternal age and BMI status. Women were stratified as younger than 35y or 35y and older, and non-obese (healthy BMI) and obese.

**Prolactin (PRL):** In SCOPE, compared to younger non-obese women, younger obese women had 26% (95% CI: 11 to 38%) lower PRL (Figure 6, Table 7). In STOP, compared to younger non-obese women, younger obese women had 24% (95% CI: 11 to 35%) lower PRL, whereas older non-obese and older obese women had 33% (95% CI: 5 to 53%) and 48 % (95% CI: 6 to 71%) lower PRL, respectively (Figure 6, Table 7).

Compared to younger non-obese women in SCOPE, younger non-obese women in STOP had 17% higher PRL (95% CI: 3 to 33%). Interestingly, compared to older obese women in SCOPE, older obese women in STOP had 48% lower PRL (95% CI: 4 to 72%) (Figure 6, Table 7).

**Figure 7:**
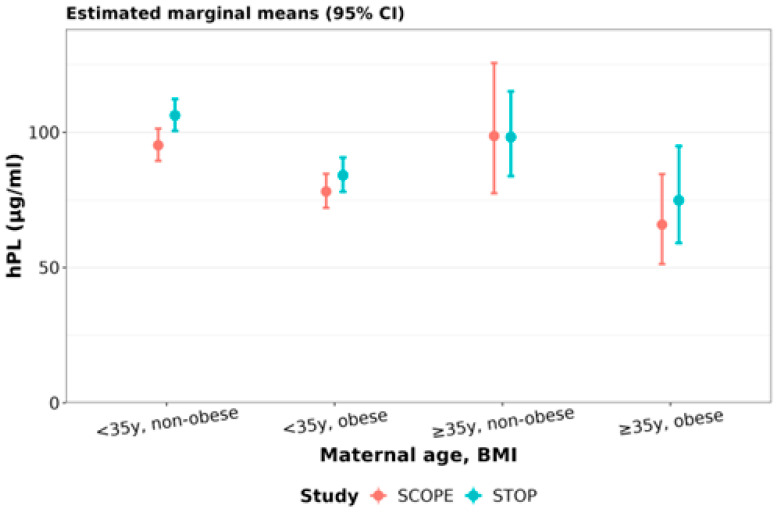
Human placental lactogen (hPL) concentrations stratified by maternal age and BMI: hPL in early gestation SCOPE (N = 465) and STOP (N = 684) women was stratified by maternal age and BMI status. Women were stratified as younger than 35y or 35y and older, and non-obese (healthy BMI) and obese.

**Human placental lactogen (hPL):** In SCOPE, compared to younger non-obese women, younger obese and older obese women had 18% (95% CI: 10 to 25%) and 31% (95% CI: 4 to 50%) lower hPL, respectively (Figure 7, Table 8). In STOP, compared to younger non-obese women, younger obese women had 21% (95% CI: 14 to 27%) lower hPL, whereas older obese women had 30% (95% CI: 4 to 48%) lower hPL (Figure 7, Table 8). Compared to younger non-obese women in SCOPE, younger non-obese women in STOP had 12% higher hPL (95% CI: 4 to 20%) (Figure 7, Table 8).

**Figure 8:**
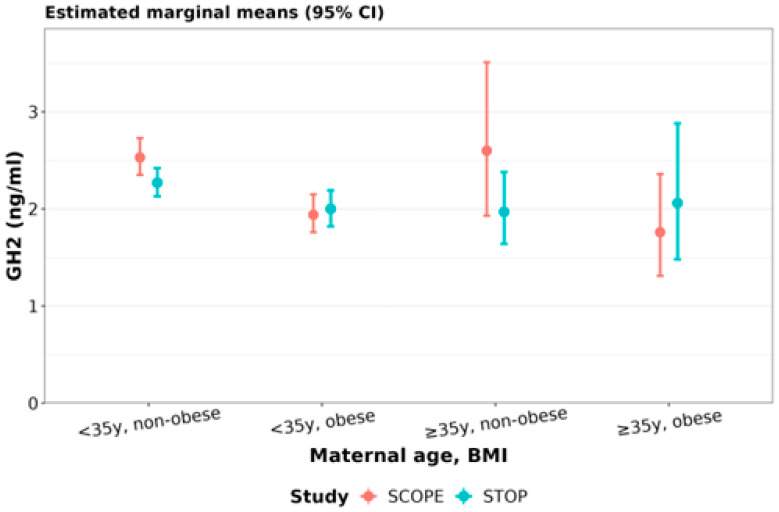
Placental growth hormone (GH2) concentrations stratified by maternal age and BMI: GH2 in early gestation SCOPE (N = 465) and STOP (N = 684) women was stratified by maternal age and BMI status. Women were stratified as younger than 35y or 35y and older and as non-obese (healthy BMI) or obese.

**Placental growth hormone (GH2):** In SCOPE, compared to younger non-obese women, younger obese women had 23% (95% CI: 13 to 32%) lower GH2 (Figure 8, Table 9). In STOP, compared to younger non-obese women, younger obese women had 12% (95% CI: 2 to 21%) lower GH2 (Figure 8, Table 9). Compared to younger non-obese women in SCOPE, younger non-obese women in STOP had 10% lower GH2 (95% CI: 2 to 18%) (Figure 8, Table 9).

## 4. Discussion

A combination of widespread FA food fortification in Australia and high FA supplementation compliance during pregnancy resulted in a considerably higher intake of daily FA than expected, as suggested by a recent review [7]. Indeed, our pregnancy data shows a significant increase in serum folate (marker of recent dietary intake) and a threefold increase in mean red cell folate (RCF; marker of long-term folate stores) in women post-FA fortification, regardless of whether women supplemented with FA in pregnancy. These findings suggest that diet-derived folate is the main driver behind high maternal folate in women post-FA food fortification. Folate deficiency, notable in women prior to FA food fortification, was mostly resolved post-FA fortification, with less than 1% of women found to be deficient. This is in line with pre and post-FA food fortification reports from the USA [62]. However, most women in our post-FA fortification cohort had red cell folate (RCF) above the normal RCPA reference range (1400 nmol/L). This is not the first report to implicate FA food fortification with folate concentrations above the normal reference range [63]. The post-FA fortification mean RCF level in our study is a conservative estimate, given that many women had RCF at the upper limit of the assay detection (1700 nmol/L). This observation is of potential concern given the lack of evidence for the safety of excess maternal folate. Clearly, not all excess folate/FA is simply excreted. 

Despite the effectiveness of FA food fortification, compliance to FA supplementation in pregnancy was higher in women post-FA fortification. Furthermore, more women post-FA fortification consumed 800 μg (or higher) daily FA supplements compared to pre-FA fortification. Prior to FA food fortification, maternal serum folate in early pregnancy increased with increasing doses of FA supplementation. However, post-FA food fortification, supplementation with FA during pregnancy did not change maternal serum or RCF levels. The utility of FA supplementation to increase maternal folate during early pregnancy in women post-Australian FA food fortification is thereby questionable, warranting re-evaluation of the paradigm that all women should supplement with FA prior to and during pregnancy in the era of widespread FA food fortification. 

Increased multivitamin supplementation and the addition of B12 to many voluntarily FA-fortified food products could also explain higher B12 in STOP. Due to their roles in the methionine cycle, ref. [64] and the observed clinical effects of combined FA and B12 supplementation, ref. [65,66,67] increased circulating folate, and B12 would be expected to lower homocysteine in women post-FA fortification. Unexpectedly, Hcy was higher in STOP women. This may be explained by the relationship between folate and B12. Despite higher B12 in STOP, the ratio of Folate:B12 is significantly higher, suggesting a disproportionate increase in circulating folate in women post-FA fortification. Both folate and B12 are key players in 1C metabolism, with B12 being essential for lowering homocysteine [68]. Higher intakes of FA relative to B12 could lead to a pseudo folate-deficient state [69,70]. This would result in a shift from the methionine to the folate pathway (left to right shift in Figure 1), favouring DNA synthesis at the expense of methylation and epigenetic programming. This shift leads to an increase in homocysteine [44,70], as we have demonstrated in the post-FA fortification cohort (STOP). Homocysteine is proinflammatory and associated with increased insulin resistance. This association is partly due to its ability to alter glucose transporter translocation to the plasma membrane, thereby reducing systemic glucose uptake, and partly due to its ability to inhibit pro-insulin receptor cleavage, causing insulin resistance [71]. Thus, higher Folate:B12 ratios and higher homocysteine levels in women post-FA fortification could promote systemic inflammation and insulin resistance. Unfortunately, we were unable to collect fasting blood to measure maternal insulin.

Nevertheless, given the role of these 1Cm variables in methylation and control of gene expression [72], increased FA intake and Folate:B12 status in women post-FA fortification could affect their systemic gene expression and, thereby, various tissue functions [69,73,74,75,76,77]. We sought to establish if peptide hormones secreted by the placenta, uterine decidua and the pituitary, which regulate insulin resistance and glucose metabolism in pregnancy, are altered with high FA intake and in women post-FA fortification. To the best of our knowledge, our work is the first to report an association between FA/folate and the secreted hormones prolactin, human placental lactogen and placental growth hormone.

Due to gestational age effects on circulating maternal hormone concentrations measured in this study, all hormone data were adjusted for gestational age at sampling, thereby enabling comparison between SCOPE and STOP. Our novel findings show lower hPL and GH2 in the first trimester of pregnancy, followed by higher PRL and hPL early in the second trimester in women post-FA fortification. GH2 promotes maternal insulin resistance [50,51], whereas PRL promotes insulin secretion to compensate for the increase in maternal insulin resistance during pregnancy [55]. hPL plays a dual role in regulating maternal glucose homeostasis: due to its high binding affinity to PRL receptors on pancreatic beta cells, it is believed to primarily promote insulin secretion [78,79], but due to its lower binding affinity to growth hormone receptors, it can also promote insulin resistance [56]. The role of hPL as driver of insulin resistance was demonstrated a few decades ago [80]. However, more recent studies clearly demonstrate its primary role in human insulin secretion [53,54]. The ambiguity of hPL action combined with the lack of insulin data makes it difficult to understand the implications of altered hPL on insulin resistance in women post-FA fortification. Lower GH2 and hPL in STOP could imply reduced insulin resistance in the first trimester. However, if hPL does indeed primarily promote insulin secretion, then it is plausible that lower hPL in early pregnancy in STOP reflects a decreased demand for insulin, which is possibly due to lower GH2. Interestingly, we found significantly higher hPL and PRL concentrations in women in early second trimester of pregnancy post-FA fortification. Depending on the biological role of hPL, this observation can have two implications. The first is that women post-FA food fortification are secreting more insulin. Although we observed no changes in GH2 (promoter of insulin resistance), Folate:B12 ratio and Hcy were higher in STOP. Given that both are associated with increased insulin resistance and GDM (and consistent with higher GDM incidence in STOP) [33,36,37,38,81,82], higher PRL and hPL may be essential to promote insulin secretion in response to cues brought on by alterations in 1C metabolism. The second possibility is that increased hPL in STOP women implies increased insulin resistance; thus, the concurrent increase in PRL may serve to promote insulin secretion and compensate for increased hPL. Whatever the metabolic implications of these hormones may be in pregnancy, their levels in the first trimester and early in the second trimester are significantly different in women post-FA fortification, and thus, they are likely to affect maternal glucose homeostasis.

To further explore the association between FA and pregnancy hormones, we examined the relationships between FA supplementation during pregnancy and hormone concentrations. Women were stratified into different FA supplementation categories depending on FA supplementation doses during early pregnancy. Strata were based on the recommended 400 μg and the most popular pregnancy brand in Australia, which contains 800 μg FA. Consistent with observed higher PRL in women post-FA food fortification, we show a FA supplementation dose effect on PRL in SCOPE, where PRL increased with increasing doses of FA supplementation. This would suggest that, in women prior to FA food fortification, FA may have had a protective effect against insulin resistance by promoting PRL secretion. However, this dose response was not observed in women post-FA food fortification. In fact, PRL was the lowest in women who supplemented with the highest dose of FA in STOP. Impairment of 1C metabolism due to long-term exposure to FA may explain the observed hormonal discrepancies between SCOPE and STOP, particularly the lower PRL in STOP women who supplemented with the highest daily dose of FA. Continual exposure to dietary FA excess, as is the case for many STOP women, combined with high FA supplementation in pregnancy can lead to alterations in 1C metabolism, particularly in the methionine cycle. Increased methylation and epigenetic metastability with FA and B12 supplementation were established in animal models, highlighting potential deleterious effects on the establishment of epigenetic gene regulation in humans [83]. High FA intake can also cause 1C metabolic enzyme saturation and a pseudo-5-methylTHF deficiency [84,85] affecting methionine cycle potential. Increased Hcy in STOP could represent an impairment of methylation potential, which would affect gene expression profiles, and thus, it would also affect PRL, hPL and GH2 secretion in women post-FA fortification.

Despite higher hPL in women post-FA fortification, FA supplementation during early pregnancy was not associated with hPL nor with GH2 in either cohort. Thus, the effects of FA on placenta-derived hPL and GH2 appear to be a result of long-term, rather than short-term, exposure to high levels of FA. This suggests that placental regulation of insulin resistance is mostly affected by long-term exposure to FA. This would explain the tripling in GDM incidence without a similar trend in magnitude in type 2 diabetes incidence, as insulin resistance in pregnancy is predominantly under placental control. In support of this contention are reports that long-term (≥3 months) FA supplementation prior to or during pregnancy are associated with a significantly higher risk of GDM compared to short-term (<3 months) FA exposure [86,87,88].

PRL, hPL and GH2 hormones play balancing roles in maternal metabolic adaptations required for a healthy pregnancy. Thus, changes to hormone concentrations post-FA fortification could foster increased insulin resistance in healthy pregnancy, placing women at an increased risk of GDM, particularly if combined with other known GDM risk factors such as increased maternal age and obesity. Advanced maternal age was shown to affect concentrations of several placental hormones across gestation [89,90]. Although there was no association between maternal age and measured hormones in SCOPE, women 35y and older had lower PRL than their younger counterparts post-FA fortification. This suggests that increased FA intake may impair the ability of older pregnant women to secrete adequate insulin to counteract the natural progression of insulin resistance in pregnancy. Unsurprisingly, the same women (35y and older) also had higher folate status, which is in line with our observations in STOP that showed lower PRL in women with highest FA intake.

We further report an association between maternal obesity and measured hormones. Overall, maternal obesity is associated with reduced hormone concentrations. This suggests that obesity has the potential to affect essential maternal adaptations required for glucose homeostasis, regardless of folate status. This is consistent with reports of reduced hPL and other hormones in pregnant women with obesity [91,92]. Interestingly, GH2, which promotes insulin resistance, was lower in obese women in SCOPE (in line with lower PRL and hPL) but not in STOP. This suggests that maternal obesity post-FA fortification may increase the risk of insulin resistance due to low PRL and hPL in the setting of higher GH2.

The combined effects of maternal age and obesity on pregnancy hormones show that younger women with a healthy BMI had significantly higher PRL and hPL and lower GH2 in STOP compared to SCOPE. However, older obese women had significantly lower PRL in STOP compared to SCOPE. Despite overall PRL being significantly higher in STOP than SCOPE, lower PRL in older and obese women in STOP indicates that women with a combination of increased maternal age and obesity post-FA fortification may now be more at risk of failure to adequately adapt to the metabolic demands of pregnancy.

**What are the potential implications of our findings for GDM?** Our understanding of the effects of studied hormones on maternal glucose homeostasis in human pregnancy is indirect, as most of our knowledge is derived from studies on rodents with a different placental lactogen-prolactin gene evolution profile. The associations between these hormones and insulin resistance or GDM are not straightforward. For example, despite strong support for the role of PRL in promoting insulin secretion in pregnancy, several studies show that high levels of PRL are associated with insulin resistance and/or hyperglycaemia, beta cell dysfunction and risk of GDM [93,94,95,96]. The effect of hPL on GDM is still not well-established, although higher hPL is associated with pre-gestational diabetes [97] and reported in amniotic fluid [98] as well as maternal blood from women with GDM [99,100]. GH2 is mostly not associated with GDM, although one study showed increased placental GH2 mRNA in pregnancies affected by GDM [101]. In clinical studies, changes in placental hormones do not directly correlate with changes in insulin resistance [102]. This implies that a complex synergistic relationship between placental hormones and other pregnancy-related factors, such as those relating to 1C metabolism, may hold a key to understanding insulin resistance in pregnancy. Emerging evidence increasingly associates high FA intake and high maternal folate status with insulin resistance and/or GDM (reviewed in Williamson et al., 2022 [34]). Many studies have identified a maternal B vitamin imbalance (high Folate:B12 ratio) as a risk factor for GDM [36,37,103]. High B12 is reported to be protective against GDM, [37,104] whereas low B12 causes accumulation of lipids in adipocytes and is associated with obesity, insulin resistance and GDM [104,105].

The novelty of our study lies with pregnancy hormones. However, with the aim to establish what has changed in healthy pregnancy after FA food fortification that may place women at increased risk of GDM, we also evaluated changes in known risk factors such as maternal age, BMI and ethnicity. Our findings suggest that these are unlikely to be adding to the increased risk of GDM in STOP. Women 35y and older are at higher risk of developing pregnancy complications, including GDM. Although the median maternal age was slightly higher in STOP, both cohorts comprised predominantly young women in their 20s with only a fraction of women (around 2%) in the greater than 35y category, and this fraction was not different between the two cohorts. BMI was also not different between SCOPE and STOP women who had an uncomplicated pregnancy. A modest change in ethnicity was observed in STOP to include a higher percentage of women of Asian and Middle Eastern decent, who are known to be at higher risk of pregnancy complications. Despite these modest changes, it is important to note that other pregnancy complications associated with increased maternal age, obesity and ethnicity are not higher in STOP nor on the same trajectory as GDM nationally. Perturbed hormone profiles interacting with FA fortification and supplementation in women with healthy pregnancies point to the strong need to examine these parameters in women who later developed GDM.

In summary, FA food fortification has resulted in significantly higher maternal serum and red cell folate concentrations, with the latter exceeding the RCPA reference range for normal folate status. FA supplementation in pregnancy does not appear to contribute to folate status in women post-FA food fortification mandate, warranting the re-evaluation of clinical guidelines. Pregnant women post-FA fortification have increased Folate:B12 ratios and homocysteine levels as well as altered levels of the hormones responsible for the regulation of glucose homeostasis. This may place them at increased risk of aberrant glucose tolerance in pregnancy. Of mention are women with increased maternal age and/or BMI, who may be at an even higher risk of inadequate adaptation to the metabolic demands of pregnancy due to lower PRL post-FA fortification mandate. Whatever the biological implications of measured hormones in this study (i.e., whether they act systemically to induce insulin resistance or via the pancreas to promote beta cell expansion and insulin secretion), the fact remains that the concentrations of these hormones were altered post-FA fortification, warranting further studies to determine biological significance. 

**Study strengths and limitation:** Our study presents findings from extensive databases and biobanks relating to two large pregnancy cohorts with similar demographics on either side of the FA food fortification mandate. These rich datasets enabled thorough evaluation of changes in pregnancy health before and after the FA food fortification mandate in women with uncomplicated pregnancies. The cohorts were highly relevant for our study, as the incidence of GDM in these cohorts increased from 5% (SCOPE) to 15% (STOP), which is in line with the national GDM rise (GDM diagnosis in SCOPE and STOP was achieved using the same current diagnostic criteria). Our study has an important limitation. Data presented are up to 16 weeks of gestation, which is early in the trajectory of increasing hormone secretion associated with insulin resistance and glucose tolerance in pregnancy. It would be invaluable to expand our findings beyond this point and to determine FA’s effect on hormones across gestation as well as to expand studies to the effects of FA on all aspects of metabolic regulation in pregnancy.

## 5. Conclusions

We have clearly shown that mandatory food fortification has significantly increased maternal folate status in early human pregnancy to beyond the normal reference range. For the first time, we provide in vivo evidence of perturbed maternal and placental endocrine status with high levels of FA consumption by the mother. These perturbations may be exacerbated in older women and those with obesity. Considering widespread global mandatory FA food fortification, as well as high FA supplementation compliance during pregnancy, particularly in Australia, and the increasing trends of delayed childbearing and maternal obesity, our findings are concerning. Further research to evaluate the effects of high FA on overall maternal endocrinology, placental function and pregnancy health, including with respect to GDM and other pregnancy complications, is urgently required.

## Figures and Tables

**Table 1:** Maternal characteristics measured in early gestation for SCOPE (14–16 weeks of gestation) and STOP (6–16 weeks) women and term pregnancy and birth outcome data for those with uncomplicated pregnancies.

	SCOPE (N = 604)	STOP (N = 711)	*p*-Value
Maternal age (y): Median (IQ range)	23.0 (20.0–26.0)	25.0 (22.0–29.0)	<0.0001
Maternal BMI: Median (IQ range)	25.1 (22.0–29.1)	25.3 (22.4–29.5)	0.27
* SEI: Median (IQ range)	24.0 (20.0–30.0)	29.0 (22.0–45.0)	<0.0001
Ethnicity: White N (%)			0.0005
Yes	555 (91.9)	595 (83.7)	
No	49 (8.1)	116 (16.3)	
Smoking: N (%)			0.002
Missing	0 (0.0)	6 (0.8)	
No	476 (78.8)	602 (84.7)	
Yes	128 (21.2)	103 (14.5)	
Folic acid supplementation (μg/day): N (%)			<0.0001
Missing	0 (0.0)	87 (12.2)	
None	122 (20.2)	49 (6.9)	
≤400	66 (10.9)	7 (1.0)	
>400 to <800	269 (44.5)	127 (17.9)	
≥800	147 (24.3)	441 (62.0)	
B12 supplementation (μg/day): N (%)			<0.0001
None	284 (47)	202 (28.4)	
Yes	320 (53)	509 (71.6)	
Blood biochemistry (** 1C metabolism)			
Serum folate (nmol/L): Median (IQ range)	36.4 (28.5–43.0)	40.5 (36.4–45.2)	<0.0001
B12 (pmol/L): Median (IQ range)	248.0 (195.5–316.0)	277.5 (219.0–355.0)	<0.0001
Homocysteine (μmol/L): Mean (SD)	4.6 (1.1)	5.0 (1.1)	<0.0001
Induction of labour: N (%)			<0.0001
No	470 (77.8)	415 (58.4)	
Yes	132 (22.2)	296 (41.6)	
Birth Outcomes			
Baby Sex: N (%)			0.55
Missing	0 (0.0)	1 (0.1)	
Female	313 (51.8)	355 (49.9)	
Male	291 (48.2)	355 (49.9)	
Gestational age at birth (weeks): Median (IQ range)	40.3 (39.4–41.0)	40.0 (39.1–40.9)	0.01
Birthweight (g): Median (IQ range)	3543 (3290–3825)	3506 (3250–3786)	0.03
Birthweight customised centile: Mean (SD)	54.2 (25.1)	53.3 (26.1)	0.52

* SEI = New Zealand Socio-Economic Index, a scale of 10–90 with a lower score indicating greater disadvantage [61]; ** 1C metabolism = one-carbon metabolism.

**Table 2:** Blood biochemistry measures of one-carbon metabolism variables in SCOPE (14–16 weeks of gestation) and STOP (6–16 weeks) women with uncomplicated pregnancies according to daily FA supplementation categories.

		Folate (nmol/L) Median (IQ Range)	B12 (pmol/L) Median (IQ Range)	Folate:B12 Ratio Median (IQ Range)	Homocysteine (μmol/L) Mean (SD)
SCOPE	None (N = 122)	27.1 (18.5–34.3)	217.5 (189.0–280.5)	117.7 (80.2–162.5)	5.1 (1.4)
	≤400 (N = 66)	35.6 (28.9–41.6)	251 (192.5–338.0)	131.6 (107.4–184.5)	4.5 (1.1)
	>400 to <800 (N = 269)	38.4 (30.4–44.2)	252 (198.0–318.0)	146.9 (107.8–228.4)	4.5 (1.1)
	≥800 (N = 147)	39.3 (34.8–44.2)	260 (208.0–315.5)	156.5 (115.7–238.9)	4.3 (0.9)
STOP	None (N = 49)	38.9 (34.3–43.2)	277.5 (219.2–316.5)	137.7 (109.8–211.8)	5.2 (1.4)
	≤400 (N = 7)	42.1 (36.6–56.3)	261 (203.5–315.5)	180.2 (165.0–201.9)	5.1 (1.1)
	>400 to <800 (N = 127)	39.8 (35.4–44.5)	260 (205.0–346.5)	168 (116.3–248.2)	5.3 (1.3)
	≥800 (N = 441)	41.3 (37.3–55.2)	281 (231.0–370.0)	158.1 (118.6–222.1)	4.8 (1.0)

**Table 3:** Serum hormone concentrations in SCOPE (14–16 weeks of gestation) and STOP (6–16 weeks) women with uncomplicated pregnancies stratified by FA supplementation categories.

Study	FA Supplementation	PRL Estimated Marginal Mean (ng/mL): (SE)	hPL Estimated Marginal Mean (μg/mL): (SE)	GH2 Estimated Marginal Mean (ng/mL): (SE)
SCOPE	None (N = 122)	71.12 (6.89)	90.59 (3.87)	2.36 (0.12)
	≤400 (N = 66)	88.11 (9.58)	94.1 (4.89)	2.57 (0.15)
	>400 to <800 (N = 269)	96.52 (8.81)	92.3 (3.4)	2.43 (0.10)
	≥800 (N = 147)	109.84 (10.75)	92.4 (3.84)	2.53 (0.13)
STOP	None (N = 49)	124.42 (15.42)	110.09 (6.22)	2.59 (0.19)
	≤400 (N = 7)	175.23 (45.83)	127.63 (16.72)	1.90 (0.34)
	>400 to <800 (N = 127)	151.39 (14.13)	107.24 (4.19)	2.29 (0.10)
	≥800 (N = 441)	90.05 (7.63)	104.86 (3.33)	2.21 (0.08)

**Table 4:** Effects of B12, Folate:B12 and Hcy on serum hormones concentrations in SCOPE (14–16 weeks of gestation) and STOP (6–16 weeks) women combined. Effect for every 100 unit increase in B12 and Folate:B12, and for every 1 unit increase in Hcy.

	B12 Ratio of Geometric Means (95% CI)	*p*-Value	Folate:B12 Ratio of Geometric Means (95% CI)	*p*-Value	Hcy Ratio of Geometric Means (95% CI)	*p*-Value
Prolactin	1.05 (1.02, 1.09)	0.0005	1.01 (0.99, 1.03)	0.4	0.93 (0.90, 0.96)	<0.0001
hPL	1.02 (1.00, 1.04)	0.03	0.99 (0.98, 1.01)	0.3	0.98 (0.97, 1.00)	0.06
GH2	1.03 (1.01, 1.05)	0.008	0.98 (0.96, 0.99)	0.004	0.98 (0.96, 1.00)	0.1

**Table 5:** Ratio of geometric means (95% CI) for different serum hormone concentrations in SCOPE and STOP stratified by maternal age.

Study	Contrast	PRL	*p*-Value	hPL	*p*-Value	GH2	*p*-Value
SCOPE	≥35y/<35y	1.11 (0.83, 1.50)	0.5	0.92 (0.77, 1.10)	0.4	0.93 (0.75, 1.14)	0.5
STOP	≥35y/<35y	0.69 (0.55, 0.87)	0.002	0.92 (0.80, 1.05)	0.2	0.93 (0.79, 1.09)	0.4

**Table 6:** Ratio of geometric means (95% CI) for different serum hormone concentrations in SCOPE and STOP stratified by maternal obesity status.

Study	Contrast	PRL	*p*-Value	hPL	*p*-Value	GH2	*p*-Value
SCOPE	Obese/Non-obese	0.81 (0.67, 0.98)	0.02	0.79 (0.72, 0.88)	<0.0001	0.73 (0.64, 0.83)	<0.0001
STOP	Obese/Non-obese	0.70 (0.59, 0.82)	<0.0001	0.77 (0.71, 0.84)	<0.0001	0.92 (0.82, 1.03)	0.2

**Table 7:** Post hoc comparisons for Prolactin (PRL) in different maternal age and BMI categories within SCOPE and STOP and between different maternal age and BMI categories between SCOPE and STOP.

Subgroup	Contrast	Ratio of Geometric Means (95% CI)	*p*-Value
SCOPE	<35y obese/<35y non-obese	0.74 (0.62, 0.89)	<0.0001
	≥35y non-obese/<35y non-obese	0.78 (0.45, 1.37)	0.7
	≥35y obese/<35y non-obese	1.17 (0.68, 2.01)	0.9
	≥35y non-obese/<35y obese	1.05 (0.59, 1.88)	1
	≥35y obese/<35y obese	1.58 (0.92, 2.72)	0.1
	≥35y obese/≥35y non-obese	1.50 (0.69, 3.25)	0.5
STOP	<35y obese/<35y non-obese	0.76 (0.65, 0.89)	<0.0001
	≥35y non-obese/<35y non-obese	0.67 (0.47, 0.95)	0.02
	≥35y obese/<35y non-obese	0.52 (0.29, 0.94)	0.02
	≥35y non-obese/<35y obese	0.88 (0.61, 1.27)	0.8
	≥35y obese/<35y obese	0.68 (0.37, 1.24)	0.4
	≥35y obese/≥35y non-obese	0.77 (0.39, 1.53)	0.8
STOP/SCOPE	<35y and non-obese	1.17 (1.03, 1.33)	0.01
	<35y and obese	1.20 (0.99, 1.46)	0.06
	≥35y and non-obese	1.00 (0.60, 1.67)	1
	≥35y and obese	0.52 (0.28, 0.96)	0.04

**Table 8:** Post hoc comparisons for human placental lactogen (hPL) in different maternal age and BMI categories within SCOPE and STOP and between different maternal age and BMI categories between SCOPE and STOP.

Subgroup	Contrast	Ratio of Geometric Means (95% CI)	*p*-Value
SCOPE	<35y obese/<35y non-obese	0.82 (0.75, 0.90)	<0.0001
	≥35y non-obese/<35y non-obese	1.04 (0.76, 1.42)	1
	≥35y obese/<35y non-obese	0.69 (0.50, 0.96)	0.02
	≥35y non-obese/<35y obese	1.26 (0.92, 1.74)	0.2
	≥35y obese/<35y obese	0.84 (0.61, 1.17)	0.5
	≥35y obese/≥35y non-obese	0.67 (0.43, 1.04)	0.09
STOP	<35y obese/<35y non-obese	0.79 (0.73, 0.86)	<0.0001
	≥35y non-obese/<35y non-obese	0.92 (0.76, 1.13)	0.7
	≥35y obese/<35y non-obese	0.70 (0.52, 0.96)	0.02
	≥35y non-obese/<35y obese	1.17 (0.95, 1.44)	0.2
	≥35y obese/<35y obese	0.89 (0.65, 1.22)	0.8
	≥35y obese/≥35y non-obese	0.76 (0.53, 1.10)	0.2
STOP/SCOPE	<35y and non-obese	1.12 (1.04, 1.20)	0.002
	<35y and obese	1.08 (0.97, 1.19)	0.1
	≥35y and non-obese	1.00 (0.75, 1.33)	1
	≥35y and obese	1.14 (0.81, 1.60)	0.5

**Table 9:** Post hoc comparisons for placental growth hormone (GH2) in different maternal age and BMI categories within and between SCOPE and STOP.

Subgroup	Contrast	Ratio of Geometric Means (95% CI)	*p*-Value
SCOPE	<35y obese/<35y non-obese	0.77 (0.68, 0.87)	<0.0001
	≥35y non-obese/<35y non-obese	1.03 (0.70, 1.51)	1
	≥35y obese/<35y non-obese	0.69 (0.47, 1.01)	0.06
	≥35y non-obese/<35y obese	1.34 (0.90, 2.00)	0.2
	≥35y obese/<35y obese	0.90 (0.61, 1.33)	0.9
	≥35y obese/≥35y non-obese	0.68 (0.39, 1.16)	0.2
STOP	<35y obese/<35y non-obese	0.88 (0.79, 0.98)	0.02
	≥35y non-obese/<35y non-obese	0.87 (0.68, 1.10)	0.4
	≥35y obese/<35y non-obese	0.91 (0.59, 1.41)	0.9
	≥35y non-obese/<35y obese	0.99 (0.76, 1.28)	1
	≥35y obese/<35y obese	1.03 (0.66, 1.61)	1
	≥35y obese/≥35y non-obese	1.05 (0.64, 1.71)	1
STOP/SCOPE	<35y and non-obese	0.90 (0.82, 0.98)	0.01
	<35y and obese	1.03 (0.90, 1.17)	0.7
	≥35y and non-obese	0.76 (0.53, 1.08)	0.1
	≥35y and obese	1.17 (0.76, 1.83)	0.5

## Data Availability

Data that support the findings of this study are openly available in Flinders University Repository of Open Access Data Sets (ROADS) at http://doi.org/10.25451/flinders.21721397, reference number 10.25451/flinders.21721397. Accessed date 13 December 2022. All protocols and models related to this project are available upon reasonable written request to the corresponding authors.

## Supplementary Materials

The following supporting information can be downloaded at: https://www.mdpi.com/article/10.3390/nu15071553/s1, Figure S1: Gestational modelling of pregnancy hormones: Serum hormone (PRL, hPL and GH2) concentrations across gestation in SCOPE and STOP women with uncomplicated pregnancies. SCOPE samples (N = 22) collected at both 11–13 and 14–16 weeks of gestation were used to estimate hormone changes across early gestation using linear mixed effects models. GA: gestational age.

## References

[1] Molloy A.M., Kirke P.N., Brody L.C., Scott J.M., Mills J.L. (2008). Effects of folate and vitamin B12 deficiencies during pregnancy on fetal, infant, and child development. Food Nutr. Bull..

[2] Field M.S., Stover P.J. (2018). Safety of folic acid. Ann. N. Y. Acad. Sci..

[3] MRC-Vitamin-Study-Research-Group (1991). Prevention of neural tube defects: Results of the Medical Research Council Vitamin Study. MRC Vitamin Study Research Group. Lancet.

[4] Czeizel A.E., Dudas I. (1992). Prevention of the first occurrence of neural-tube defects by periconceptional vitamin supplementation. N. Engl. J. Med..

[5] Murphy M.E., Westmark C.J. (2020). Folic Acid Fortification and Neural Tube Defect Risk: Analysis of the Food Fortification Initiative Dataset. Nutrients.

[6] Food-Standards-Australia-and-New-Zealand Folic Acid/Folate and Pregnancy. https://www.foodstandards.gov.au/consumer/nutrition/folicmandatory/Pages/default.aspx.

[7] Ledowsky C., Mahimbo A., Scarf V., Steel A. (2022). Women Taking a Folic Acid Supplement in Countries with Mandatory Food Fortification Programs May Be Exceeding the Upper Tolerable Limit of Folic Acid: A Systematic Review. Nutrients.

[8] Livock M., Anderson P.J., Lewis S., Bowden S., Muggli E., Halliday J. (2017). Maternal micronutrient consumption periconceptionally and during pregnancy: A prospective cohort study. Public. Health Nutr..

[9] Pietrzik K., Bailey L., Shane B. (2010). Folic acid and L-5-methyltetrahydrofolate: Comparison of clinical pharmacokinetics and pharmacodynamics. Clin. Pharmacokinet..

[10] Jhaveri M.S., Wagner C., Trepel J.B. (2001). Impact of extracellular folate levels on global gene expression. Mol. Pharmacol..

[11] Pufulete M., Al-Ghnaniem R., Khushal A., Appleby P., Harris N., Gout S., Emery P.W., Sanders T.A. (2005). Effect of folic acid supplementation on genomic DNA methylation in patients with colorectal adenoma. Gut.

[12] Kok D.E., Dhonukshe-Rutten R.A., Lute C., Heil S.G., Uitterlinden A.G., van der Velde N., van Meurs J.B., van Schoor N.M., Hooiveld G.J., de Groot L.C. (2015). The effects of long-term daily folic acid and vitamin B12 supplementation on genome-wide DNA methylation in elderly subjects. Clin. Epigenetics.

[13] Froese D.S., Fowler B., Baumgartner M.R. (2019). Vitamin B12, folate, and the methionine remethylation cycle-biochemistry, pathways, and regulation. J. Inherit. Metab. Dis..

[14] Yasuda S., Hasui S., Yamamoto C., Yoshioka C., Kobayashi M., Itagaki S., Hirano T., Iseki K. (2008). Placental folate transport during pregnancy. Biosci. Biotechnol. Biochem..

[15] Solanky N., Requena Jimenez A., D’Souza S.W., Sibley C.P., Glazier J.D. (2010). Expression of folate transporters in human placenta and implications for homocysteine metabolism. Placenta.

[16] Williams P.J., Bulmer J.N., Innes B.A., Broughton Pipkin F. (2011). Possible roles for folic acid in the regulation of trophoblast invasion and placental development in normal early human pregnancy. Biol. Reprod..

[17] Irwin R.E., Thursby S.J., Ondicova M., Pentieva K., McNulty H., Richmond R.C., Caffrey A., Lees-Murdock D.J., McLaughlin M., Cassidy T. (2019). A randomized controlled trial of folic acid intervention in pregnancy highlights a putative methylation-regulated control element at ZFP57. Clin. Epigenetics.

[18] Ondicova M., Irwin R.E., Thursby S.J., Hilman L., Caffrey A., Cassidy T., McLaughlin M., Lees-Murdock D.J., Ward M., Murphy M. (2022). Folic acid intervention during pregnancy alters DNA methylation, affecting neural target genes through two distinct mechanisms. Clin. Epigenetics.

[19] Tserga A., Binder A.M., Michels K.B. (2017). Impact of folic acid intake during pregnancy on genomic imprinting of IGF2/H19 and 1-carbon metabolism. FASEB J..

[20] Rahat B., Hamid A., Bagga R., Kaur J. (2022). Folic Acid Levels During Pregnancy Regulate Trophoblast Invasive Behavior and the Possible Development of Preeclampsia. Front. Nutr..

[21] Ly L., Chan D., Landry M., Angle C., Martel J., Trasler J. (2020). Impact of mothers’ early life exposure to low or high folate on progeny outcome and DNA methylation patterns. Environ. Epigenet.

[22] Rosario F.J., Nathanielsz P.W., Powell T.L., Jansson T. (2017). Maternal folate deficiency causes inhibition of mTOR signaling, down-regulation of placental amino acid transporters and fetal growth restriction in mice. Sci. Rep..

[23] Furness D.L., Yasin N., Dekker G.A., Thompson S.D., Roberts C.T. (2012). Maternal red blood cell folate concentration at 10–12 weeks gestation and pregnancy outcome. J. Matern.-Fetal Neonatal Med..

[24] Bodnar L.M., Himes K.P., Venkataramanan R., Chen J.Y., Evans R.W., Meyer J.L., Simhan H.N. (2010). Maternal serum folate species in early pregnancy and risk of preterm birth. Am. J. Clin. Nutr..

[25] Hodgetts V.A., Morris R.K., Francis A., Gardosi J., Ismail K.M. (2015). Effectiveness of folic acid supplementation in pregnancy on reducing the risk of small-for-gestational age neonates: A population study, systematic review and meta-analysis. BJOG.

[26] Liu X., Lv L., Zhang H., Zhao N., Qiu J., He X., Zhou M., Xu X., Cui H., Liu S. (2016). Folic acid supplementation, dietary folate intake and risk of preterm birth in China. Eur. J. Nutr..

[27] Li B., Zhang X., Peng X., Zhang S., Wang X., Zhu C. (2019). Folic Acid and Risk of Preterm Birth: A Meta-Analysis. Front. Neurosci..

[28] Bailey S.W., Ayling J.E. (2009). The extremely slow and variable activity of dihydrofolate reductase in human liver and its implications for high folic acid intake. Proc. Natl. Acad. Sci. USA.

[29] Patanwala I., King M.J., Barrett D.A., Rose J., Jackson R., Hudson M., Philo M., Dainty J.R., Wright A.J., Finglas P.M. (2014). Folic acid handling by the human gut: Implications for food fortification and supplementation. Am. J. Clin. Nutr..

[30] Hu J., Wang B., Sahyoun N.R. (2016). Application of the Key Events Dose-response Framework to Folate Metabolism. Crit. Rev. Food Sci. Nutr..

[31] Sweeney M.R., McPartlin J., Scott J. (2007). Folic acid fortification and public health: Report on threshold doses above which unmetabolised folic acid appear in serum. BMC Public Health.

[32] Obeid R., Kirsch S.H., Dilmann S., Klein C., Eckert R., Geisel J., Herrmann W. (2016). Folic acid causes higher prevalence of detectable unmetabolized folic acid in serum than B-complex: A randomized trial. Eur. J. Nutr..

[33] Dai C., Fei Y., Li J., Shi Y., Yang X. (2021). A Novel Review of Homocysteine and Pregnancy Complications. Biomed. Res. Int..

[34] Williamson J.M., Arthurs A.L., Smith M.D., Roberts C.T., Jankovic-Karasoulos T. (2022). High Folate, Perturbed One-Carbon Metabolism and Gestational Diabetes Mellitus. Nutrients.

[35] Krishnaveni G.V., Veena S.R., Karat S.C., Yajnik C.S., Fall C.H. (2014). Association between maternal folate concentrations during pregnancy and insulin resistance in Indian children. Diabetologia.

[36] Lai J.S., Pang W.W., Cai S., Lee Y.S., Chan J.K.Y., Shek L.P.C., Yap F.K.P., Tan K.H., Godfrey K.M., van Dam R.M. (2018). High folate and low vitamin B12 status during pregnancy is associated with gestational diabetes mellitus. Clin. Nutr..

[37] Saravanan P., Sukumar N., Adaikalakoteswari A., Goljan I., Venkataraman H., Gopinath A., Bagias C., Yajnik C.S., Stallard N., Ghebremichael-Weldeselassie Y. (2021). Association of maternal vitamin B12 and folate levels in early pregnancy with gestational diabetes: A prospective UK cohort study (PRiDE study). Diabetologia.

[38] Deng M., Zhou J., Tang Z., Xiang J., Yi J., Peng Y., Di L., Zhai X., Yang M., Du Y. (2020). The correlation between plasma total homocysteine level and gestational diabetes mellitus in a Chinese Han population. Sci. Rep..

[39] Zheng Y., Deng H.Y., Qiao Z.Y., Gong F.X. (2021). Homocysteine level and gestational diabetes mellitus: A systematic review and meta-analysis. Gynecol. Endocrinol..

[40] AIHW (2020). Australia’s Health. https://www.aihw.gov.au/reports/diabetes/diabetes/contents/how-many-australians-have-diabetes/gestational-diabetes.

[41] Laurie J.G., McIntyre H.D. (2020). A Review of the Current Status of Gestational Diabetes Mellitus in Australia-The Clinical Impact of Changing Population Demographics and Diagnostic Criteria on Prevalence. Int. J. Environ. Res. Public. Health.

[42] Huang Y., He Y., Sun X., He Y., Li Y., Sun C. (2014). Maternal high folic acid supplement promotes glucose intolerance and insulin resistance in male mouse offspring fed a high-fat diet. Int. J. Mol. Sci..

[43] Keating E., Correia-Branco A., Araujo J.R., Meireles M., Fernandes R., Guardao L., Guimaraes J.T., Martel F., Calhau C. (2015). Excess perigestational folic acid exposure induces metabolic dysfunction in post-natal life. J. Endocrinol..

[44] Koseki K., Maekawa Y., Bito T., Yabuta Y., Watanabe F. (2020). High-dose folic acid supplementation results in significant accumulation of unmetabolized homocysteine, leading to severe oxidative stress in Caenorhabditis elegans. Redox Biol..

[45] Liu Z., Zhang Y., Liu Z., Tian Z., Pei X., Liu L., Li Y. (2022). Folic acid oversupplementation during pregnancy disorders lipid metabolism in male offspring via regulating arginase 1-associated NOS3-AMPKalpha pathway. Clin. Nutr..

[46] Pannia E., Yang N.V., Ho M., Chatterjee D., Hammoud R., Kubant R., Anderson G.H. (2020). Folic acid content of diet during pregnancy determines post-birth re-set of metabolism in Wistar rat dams. J. Nutr. Biochem..

[47] Stern C., Schwarz S., Moser G., Cvitic S., Jantscher-Krenn E., Gauster M., Hiden U. (2021). Placental Endocrine Activity: Adaptation and Disruption of Maternal Glucose Metabolism in Pregnancy and the Influence of Fetal Sex. Int. J. Mol. Sci..

[48] Kampmann U., Knorr S., Fuglsang J., Ovesen P. (2019). Determinants of Maternal Insulin Resistance during Pregnancy: An Updated Overview. J. Diabetes Res..

[49] Napso T., Yong H.E.J., Lopez-Tello J., Sferruzzi-Perri A.N. (2018). The Role of Placental Hormones in Mediating Maternal Adaptations to Support Pregnancy and Lactation. Front. Physiol..

[50] Barbour L.A., Shao J., Qiao L., Leitner W., Anderson M., Friedman J.E., Draznin B. (2004). Human placental growth hormone increases expression of the p85 regulatory unit of phosphatidylinositol 3-kinase and triggers severe insulin resistance in skeletal muscle. Endocrinology.

[51] Barbour L.A., Shao J., Qiao L., Pulawa L.K., Jensen D.R., Bartke A., Garrity M., Draznin B., Friedman J.E. (2002). Human placental growth hormone causes severe insulin resistance in transgenic mice. Am. J. Obstet. Gynecol..

[52] Sonagra A.D., Biradar S.M., K D., Murthy D.S.J. (2014). Normal pregnancy—A state of insulin resistance. J. Clin. Diagn. Res..

[53] Simpson S., Smith L., Bowe J. (2018). Placental peptides regulating islet adaptation to pregnancy: Clinical potential in gestational diabetes mellitus. Curr. Opin. Pharmacol..

[54] Banerjee R.R., Cyphert H.A., Walker E.M., Chakravarthy H., Peiris H., Gu X., Liu Y., Conrad E., Goodrich L., Stein R.W. (2016). Gestational Diabetes Mellitus From Inactivation of Prolactin Receptor and MafB in Islet beta-Cells. Diabetes.

[55] Brelje T.C., Scharp D.W., Lacy P.E., Ogren L., Talamantes F., Robertson M., Friesen H.G., Sorenson R.L. (1993). Effect of homologous placental lactogens, prolactins, and growth hormones on islet B-cell division and insulin secretion in rat, mouse, and human islets: Implication for placental lactogen regulation of islet function during pregnancy. Endocrinology.

[56] Ryan E.A., Enns L. (1988). Role of gestational hormones in the induction of insulin resistance. J. Clin. Endocrinol. Metab..

[57] Richard K., Holland O., Landers K., Vanderlelie J.J., Hofstee P., Cuffe J.S.M., Perkins A.V. (2017). Review: Effects of maternal micronutrient supplementation on placental function. Placenta.

[58] Baker B.C., Hayes D.J., Jones R.L. (2018). Effects of micronutrients on placental function: Evidence from clinical studies to animal models. Reproduction.

[59] Rasool A., Alvarado-Flores F., O’Tierney-Ginn P. (2021). Placental Impact of Dietary Supplements: More Than Micronutrients. Clin. Ther..

[60] Meher A., Sundrani D., Joshi S. (2015). Maternal nutrition influences angiogenesis in the placenta through peroxisome proliferator activated receptors: A novel hypothesis. Mol. Reprod. Dev..

[61] Davis P., McLeod K., Ransom M., Ongley P., Pearce N., Howden-Chapman P. (1999). The New Zealand Socioeconomic Index: Developing and validating an occupationally-derived indicator of socio-economic status. Aust. N. Z. J. Public. Health.

[62] van Gool J.D., Hirche H., Lax H., De Schaepdrijver L. (2018). Folic acid and primary prevention of neural tube defects: A review. Reprod. Toxicol..

[63] Pinunuri R., Castano E., Ronco A. (2017). Folates during Pregnancy: Food Fortification versus Supplementation with Folic Acid. Acad. J. Pediatr. Neonatol..

[64] Kalhan S.C., Marczewski S.E. (2012). Methionine, homocysteine, one carbon metabolism and fetal growth. Rev. Endocr. Metab. Disord..

[65] Azadibakhsh N., Hosseini R.S., Atabak S., Nateghiyan N., Golestan B., Rad A.H. (2009). Efficacy of folate and vitamin B12 in lowering homocysteine concentrations in hemodialysis patients. Saudi J. Kidney Dis. Transpl..

[66] Armitage J.M., Bowman L., Clarke R.J., Wallendszus K., Bulbulia R., Rahimi K., Haynes R., Parish S., Sleight P., Study of the Effectiveness of Additional Reductions in Cholesterol and Homocysteine (SEARCH) Collaborative Group (2010). Effects of homocysteine-lowering with folic acid plus vitamin B12 vs placebo on mortality and major morbidity in myocardial infarction survivors: A randomized trial. JAMA.

[67] Lee B.J., Huang M.C., Chung L.J., Cheng C.H., Lin K.L., Su K.H., Huang Y.C. (2004). Folic acid and vitamin B12 are more effective than vitamin B6 in lowering fasting plasma homocysteine concentration in patients with coronary artery disease. Eur. J. Clin. Nutr..

[68] Smith A.D., Warren M.J., Refsum H. (2018). Vitamin B(12). Adv. Food Nutr. Res..

[69] Ortbauer M., Ripper D., Fuhrmann T., Lassi M., Auernigg-Haselmaier S., Stiegler C., Konig J. (2016). Folate deficiency and over-supplementation causes impaired folate metabolism: Regulation and adaptation mechanisms in Caenorhabditis elegans. Mol. Nutr. Food Res..

[70] Maher A., Sobczynska-Malefora A. (2021). The Relationship Between Folate, Vitamin B12 and Gestational Diabetes Mellitus With Proposed Mechanisms and Foetal Implications. J. Family Reprod. Health.

[71] Zhang X., Qu Y.Y., Liu L., Qiao Y.N., Geng H.R., Lin Y., Xu W., Cao J., Zhao J.Y. (2021). Homocysteine inhibits pro-insulin receptor cleavage and causes insulin resistance via protein cysteine-homocysteinylation. Cell Rep..

[72] Salbaum J.M., Kappen C. (2012). Genetic and epigenomic footprints of folate. Prog. Mol. Biol. Transl. Sci..

[73] Haas C.B., Su Y.R., Petersen P., Wang X., Bien S.A., Lin Y., Albanes D., Weinstein S.J., Jenkins M.A., Figueiredo J.C. (2022). Interactions between folate intake and genetic predictors of gene expression levels associated with colorectal cancer risk. Sci. Rep..

[74] Chu D., Li L., Jiang Y., Tan J., Ji J., Zhang Y., Jin N., Liu F. (2019). Excess Folic Acid Supplementation Before and During Pregnancy and Lactation Activates Fos Gene Expression and Alters Behaviors in Male Mouse Offspring. Front. Neurosci..

[75] Sanchez H., Hossain M.B., Lera L., Hirsch S., Albala C., Uauy R., Broberg K., Ronco A.M. (2017). High levels of circulating folate concentrations are associated with DNA methylation of tumor suppressor and repair genes p16, MLH1, and MGMT in elderly Chileans. Clin. Epigenetics.

[76] Penailillo R., Guajardo A., Llanos M., Hirsch S., Ronco A.M. (2015). Folic acid supplementation during pregnancy induces sex-specific changes in methylation and expression of placental 11beta-hydroxysteroid dehydrogenase 2 in rats. PLoS ONE.

[77] Alnabbat K.I., Fardous A.M., Cabelof D.C., Heydari A.R. (2022). Excessive Folic Acid Mimics Folate Deficiency in Human Lymphocytes. Curr. Issues Mol. Biol..

[78] Lombardo M.F., De Angelis F., Bova L., Bartolini B., Bertuzzi F., Nano R., Capuani B., Lauro R., Federici M., Lauro D. (2011). Human placental lactogen (hPL-A) activates signaling pathways linked to cell survival and improves insulin secretion in human pancreatic islets. Islets.

[79] Baeyens L., Hindi S., Sorenson R.L., German M.S. (2016). beta-Cell adaptation in pregnancy. Diabetes Obes. Metab..

[80] Handwerger S., Freemark M. (1987). Role of placental lactogen and prolactin in human pregnancy. Adv. Exp. Med. Biol..

[81] Chen X., Zhang Y., Chen H., Jiang Y., Wang Y., Wang D., Li M., Dou Y., Sun X., Huang G. (2021). Association of Maternal Folate and Vitamin B12 in Early Pregnancy With Gestational Diabetes Mellitus: A Prospective Cohort Study. Diabetes Care.

[82] Gong T., Wang J., Yang M., Shao Y., Liu J., Wu Q., Xu Q., Wang H., He X., Chen Y. (2016). Serum homocysteine level and gestational diabetes mellitus: A meta-analysis. J. Diabetes Investig..

[83] Waterland R.A., Jirtle R.L. (2003). Transposable elements: Targets for early nutritional effects on epigenetic gene regulation. Mol. Cell. Biol..

[84] Cornet D., Clement A., Clement P., Menezo Y. (2019). High doses of folic acid induce a pseudo-methylenetetrahydrofolate syndrome. SAGE Open. Med. Case Rep..

[85] Luan Y., Leclerc D., Cosin-Tomas M., Malysheva O.V., Wasek B., Bottiglieri T., Caudill M.A., Rozen R. (2021). Moderate Folic Acid Supplementation in Pregnant Mice Results in Altered Methyl Metabolism and in Sex-Specific Placental Transcription Changes. Mol. Nutr. Food Res..

[86] Li N., Jiang J., Guo L. (2022). Effects of maternal folate and vitamin B12 on gestational diabetes mellitus: A dose-response meta-analysis of observational studies. Eur. J. Clin. Nutr..

[87] Huang L., Yu X., Li L., Chen Y., Yang Y., Yang Y., Hu Y., Zhao Y., Tang H., Xu D. (2019). Duration of periconceptional folic acid supplementation and risk of gestational diabetes mellitus. Asia Pac. J. Clin. Nutr..

[88] Cheng G., Sha T., Gao X., He Q., Wu X., Tian Q., Yang F., Tang C., Wu X., Xie Q. (2019). The Associations between the Duration of Folic Acid Supplementation, Gestational Diabetes Mellitus, and Adverse Birth Outcomes based on a Birth Cohort. Int. J. Environ. Res. Public. Health.

[89] Lean S.C., Heazell A.E.P., Dilworth M.R., Mills T.A., Jones R.L. (2017). Placental Dysfunction Underlies Increased Risk of Fetal Growth Restriction and Stillbirth in Advanced Maternal Age Women. Sci. Rep..

[90] Haavaldsen C., Fedorcsak P., Tanbo T., Eskild A. (2014). Maternal age and serum concentration of human chorionic gonadotropin in early pregnancy. Acta Obstet. Gynecol. Scand..

[91] Cattini P.A., Jin Y., Jarmasz J.S., Noorjahan N., Bock M.E. (2020). Obesity and regulation of human placental lactogen production in pregnancy. J. Neuroendocrinol..

[92] Lassance L., Haghiac M., Minium J., Catalano P., Hauguel-de Mouzon S. (2015). Obesity-induced down-regulation of the mitochondrial translocator protein (TSPO) impairs placental steroid production. J. Clin. Endocrinol. Metab..

[93] Tuzcu A., Bahceci M., Dursun M., Turgut C., Bahceci S. (2003). Insulin sensitivity and hyperprolactinemia. J. Endocrinol. Investig..

[94] Yang H., Lin J., Li H., Liu Z., Chen X., Chen Q. (2021). Prolactin Is Associated With Insulin Resistance and Beta-Cell Dysfunction in Infertile Women With Polycystic Ovary Syndrome. Front. Endocrinol..

[95] Atmaca A., Bilgici B., Ecemis G.C., Tuncel O.K. (2013). Evaluation of body weight, insulin resistance, leptin and adiponectin levels in premenopausal women with hyperprolactinemia. Endocrine.

[96] Ekinci E.I., Torkamani N., Ramchand S.K., Churilov L., Sikaris K.A., Lu Z.X., Houlihan C.A. (2017). Higher maternal serum prolactin levels are associated with reduced glucose tolerance during pregnancy. J. Diabetes Investig..

[97] Ngala R.A., Fondjo L.A., Gmagna P., Ghartey F.N., Awe M.A. (2017). Placental peptides metabolism and maternal factors as predictors of risk of gestational diabetes in pregnant women. A case-control study. PLoS ONE.

[98] Lolis D., Tzingounis V., Kaskarelis D. (1978). Maternal serums and amniotic fluid levels of human placental lactogen in gestational diabetes. Eur. J. Clin. Investig..

[99] Ursell W., Brudenell M., Chard T. (1973). Placental lactogen levels in diabetic pregnancy. Br. Med. J..

[100] Soler N.G., Nicholson H.O., Malins J.M. (1975). Serial determinations of human placental lactogen in the management of diabetic pregnancy. Lancet.

[101] Hu L., Lytras A., Bock M.E., Yuen C.K., Dodd J.G., Cattini P.A. (1999). Detection of placental growth hormone variant and chorionic somatomammotropin-L RNA expression in normal and diabetic pregnancy by reverse transcriptase-polymerase chain reaction. Mol. Cell. Endocrinol..

[102] Kirwan J.P., Hauguel-De Mouzon S., Lepercq J., Challier J.C., Huston-Presley L., Friedman J.E., Kalhan S.C., Catalano P.M. (2002). TNF-alpha is a predictor of insulin resistance in human pregnancy. Diabetes.

[103] Li S., Hou Y., Yan X., Wang Y., Shi C., Wu X., Liu H., Zhang L., Zhang X., Liu J. (2019). Joint effects of folate and vitamin B12 imbalance with maternal characteristics on gestational diabetes mellitus. Journal. Diabetes.

[104] Chen X., Du Y., Xia S., Li Z., Liu J. (2022). Vitamin B(12) and gestational diabetes mellitus: A systematic review and meta-analysis. Br. J. Nutr..

[105] Adaikalakoteswari A., Vatish M., Alam M.T., Ott S., Kumar S., Saravanan P. (2017). Low Vitamin B12 in Pregnancy Is Associated With Adipose-Derived Circulating miRs Targeting PPARgamma and Insulin Resistance. J. Clin. Endocrinol. Metab..

