# Supplementary material for: Elevated Maternal Folate Status and Changes in Maternal Prolactin, Placental Lactogen and Placental Growth Hormone Following Folic Acid Food Fortification: Evidence from Two Prospective Pregnancy Cohorts
Source: Nutrients. 2023 Mar 23;15(7):1553. doi: 10.3390/nu15071553 (PMC10097170; doi:10.3390/nu15071553)
Supplement: Supplementary file 1 [file nutrients-15-01553-s001.zip › nutrients-2244905-supplementary.pdf]

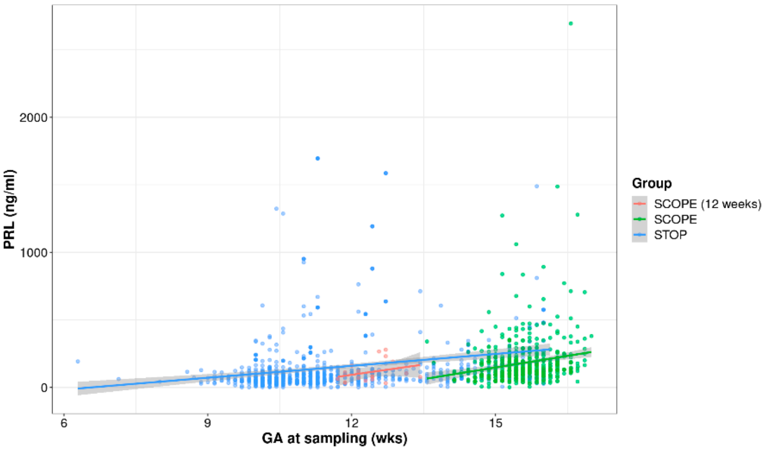

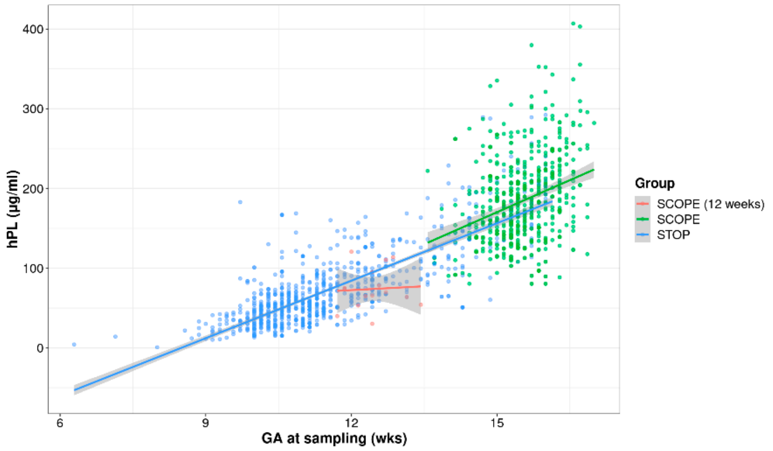

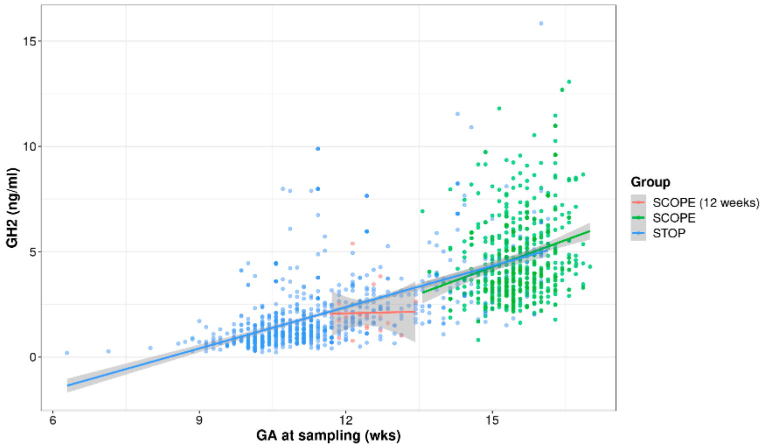

Supplementary Figure S1. Gestational modelling of pregnancy hormones: Serum hormone (PRL, hPL and GH2) concentrations across gestation in SCOPE and STOP women with uncomplicated pregnancies. SCOPE samples (N=22) collected at both 11-13 and 14-16 weeks' were used to estimate hormone changes across early gestation using linear mixed effects models. GA: gestational age.
